# Developing a therapeutic elastase that stimulates anti-tumor immunity by selectively killing cancer cells

**DOI:** 10.1016/j.xcrm.2025.102446

**Published:** 2025-11-07

**Authors:** Ravindra Gujar, Chang Cui, Maria Fumagalli, Nicole Martinez, Afshin Bahador, Alain Algazi, Kevin Harrington, Court Turner, Lev Becker

**Affiliations:** 1 Onchilles Pharma Inc., San Diego, CA, USA; 2 South Coast Gynecologic Oncology Inc, San Diego, CA, USA; 3 Helen Diller Family Comprehensive Cancer Center, Department of Medicine, University of California, San Francisco, San Francisco, CA, USA; 4 The Institute of Cancer Research and The Royal Marsden NHS Foundation Trust National Institute of Health Research Biomedical Research Centre, London, UK

**Keywords:** N17350, ELANE pathway, elastase, histone H1, cytotoxic therapeutic, abscopal effect, selective cancer killing, immunogenic cell death, immunotherapy, pan-cancer

## Abstract

Recent clinical studies highlight the effectiveness of combining cytotoxic agents with immunotherapies, emphasizing the need for next-generation treatments that integrate both therapeutic approaches. Here, we use 30 cancer cell lines, 15 tumor models, and 45 patient samples to develop N17350, a therapeutic elastase that targets the “neutrophil elastase pathway” to induce tumor regression and stimulate anti-tumor immunity. N17350 leverages linker histone H1.0 and H1.2, proteins elevated in many cancers, to trigger immunogenic cancer cell death while preserving immune cells. Intra-tumoral N17350 administration induces rapid, genotype-independent tumor regression, triggering CD8^+^ T cell activation to promote durable responses and enable checkpoint inhibitor efficacy in refractory models. N17350 maintains potency with repeated dosing and across diverse treatment histories, including resistance to chemotherapies and checkpoint inhibitors. These findings support the advancement of N17350 to first-in-human clinical trials as a cytotoxic agent designed to stimulate anti-tumor immunity by selectively killing cancer cells.

## Highlights

• N17350 selectively kills cancer cells via immunogenic cell death
• N17350 regresses tumors and stimulates anti-tumor immunity
• N17350 maintains potency following repeated dosing
• N17350 treats therapy-resistant tumors

## Graphical abstract

**Figure:**
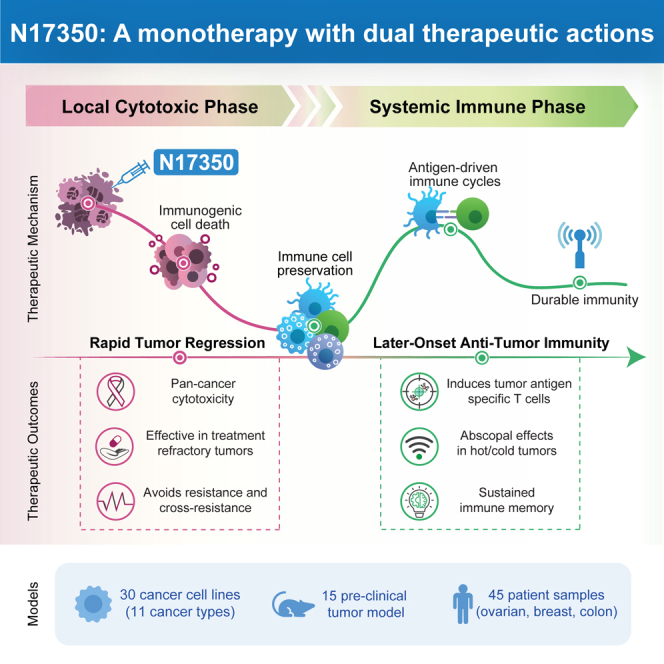

## Teaser

Gujar et al. develop N17350, a next-generation therapeutic elastase that selectively kills cancer cells, drives tumor regression, and induces systemic anti-tumor immunity. It remains effective with repeated dosing and in resistant cancers, positioning N17350 as a strong candidate for first-in-human clinical evaluation.

## Introduction

Non-surgical cancer therapies are generally classified into two primary categories: cytotoxic treatments, which directly eliminate cancer cells (e.g., chemotherapy and radiation), and immunotherapies (e.g., checkpoint inhibitors [CPIs]), which enhance the immune system’s ability to target cancer cells. These approaches can be complementary. Cytotoxic therapies can stimulate anti-tumor immunity by releasing tumor-associated antigens and immune-activating molecules during immunogenic cell death,^1^^,^^2^ which can be amplified by immunotherapies.^3^

This synergy between cytotoxic treatments and immunotherapies represents a promising cancer treatment strategy, enhancing both direct tumor cell killing and long-term immunity. In fact, patients who receive both chemotherapy and immunotherapy can experience better clinical outcomes than those treated with either modality alone.^4^^,^^5^ However, this benefit can be limited by chemotherapy’s toxicity to mature T cells,^6^^,^^7^^,^^8^ which are essential for effective anti-tumor immunity.

To improve this approach, next-generation cytotoxic agents are needed—particularly those that effectively kill cancer cells while preserving or even boosting immune cells. These agents should combine broad cancer-killing abilities to overcome tumor heterogeneity and generate a diverse antigen repertoire, all while preserving immune cells and stimulating anti-tumor immunity. This would enhance their efficacy both as standalone treatments and in combination with CPIs. However, achieving this requires a mechanism that targets cancer cells across various genetic backgrounds, while sparing normal cells—a significant challenge, as current cytotoxic agents typically achieve one goal at the expense of the other.

We recently identified neutrophil elastase (ELANE), a serine protease derived from neutrophils, as a next-generation cytotoxic agent that meets both criteria.^9^ ELANE kills a broad range of cancer cells (35 out of 35 tested) while preserving non-cancer cells, including immune cells. Its mechanism involves neuropilin-1-mediated uptake by cancer cells, proteolytic activation of the CD95 death domain, and cytosolic translocation of histone H1, which lead to DNA damage, mitochondrial dysfunction, and activation of cell death effectors—an action known as the “ELANE pathway.” Intra-tumorally delivered ELANE has demonstrated efficacy in attenuating tumor growth in 9 out of 9 pre-clinical models and has been shown to generate CD8^+^-T-cell-mediated abscopal effects in syngeneic and genetically engineered pre-clinical models.

Although ELANE’s therapeutic efficacy is improved by porcine pancreatic elastase (PPE), an ELANE ortholog that better evades protease inhibitors, clinical translation is hindered by limited efficacy in the tumor microenvironment. To overcome this limitation, we developed N17350, a therapeutic elastase optimized for intra-tumoral delivery. N17350 cleaves the C-terminus of CD95 to liberate its death domain and demonstrates improved enzymatic activity and enhanced cell uptake, both of which optimize its ability to kill cancer cells relative to PPE (Figure S1).

Here, we incorporated 30 cancer cell lines, 15 tumor models, and 45 patient samples to rigorously evaluate the therapeutic properties of N17350 in pre-clinical studies spanning melanoma, ovarian, colon, breast, esophageal, head and neck, lung, liver, pancreatic, and prostate cancers. To facilitate clinical translation, we emphasized testing in patient samples and evaluating performance with experiments designed to simulate complexities encountered in early phase clinical trials (e.g., treatment-refractory disease).

Our findings underscore the broad and powerful therapeutic properties that emerge with N17350, a cytotoxic agent developed to effectively kill cancer cells and preserve immune cells by targeting the ELANE pathway. These include prompt tumor regression across cancer types, stimulation of anti-tumor immunity across tumor immunotypes, and sustained potency following repetitive administration and across diverse treatment histories. These findings position N17350 as a promising candidate for first-in-human clinical trials as a cytotoxic agent designed to stimulate anti-tumor immunity by selectively killing cancer cells.

## Results

### N17350 exhibits pan-cancer cytotoxicity with selectivity to preserve immune cells

In principle, developing a cytotoxic agent that effectively kills cancer cells to activate anti-tumor immunity relies on two key properties: (1) genotype-independent killing to overcome tumor heterogeneity and (2) selectivity to preserve non-cancer cells including critical immune cells. Previous studies showed that these two attributes are inherent to serine proteases that target the ELANE pathway, including ELANE and PPE.^9^^,^^10^^,^^11^ We used a wide range of cell lines, pre-clinical models, and patient samples to determine whether N17350 also possesses these properties.

**Figure 1:**
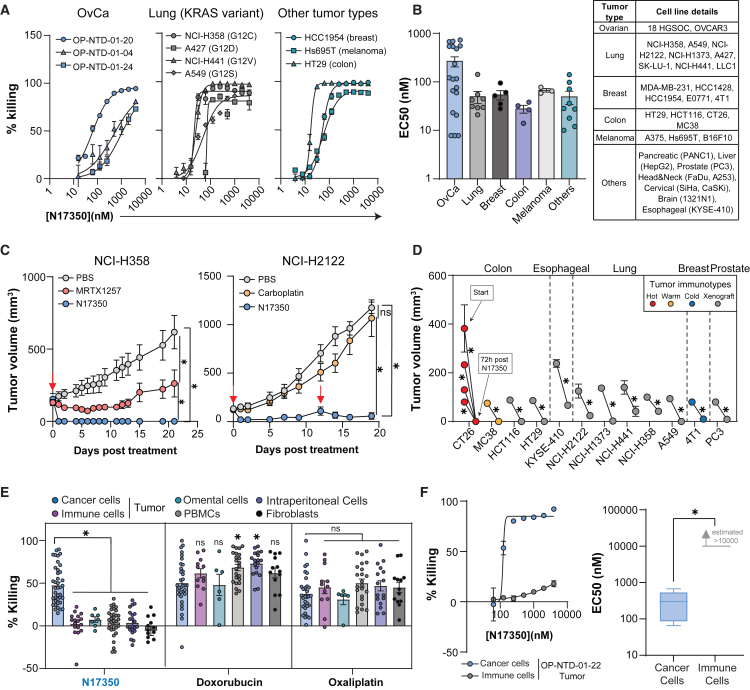
N17350 broadly kills cancer cells while preserving immune cells (A and B) Various cancer cell lines and primary cancer cells from OvCa patients were treated with increasing doses of N17350 for 24 h, and the EC50 was determined (n = 2–6/dose). (A) Representative killing curves for OvCa patient cancer cells (left), lung cancer cells with distinct KRAS variants (middle), and other tumor types (right). (B) Summary of EC50 values (left) and cancer-cell-type origin (right). (C) Representative tumor growth curve for NCI-H358 and NCI-H2122 tumors (non-small cell lung cancer) treated with N17350 (400 μg/100 mm^3^, intra-tumoral), MRTX1257 (100 mg/kg, oral, daily), or carboplatin (100 mg/kg, intraperitoneal, days 0 and 7); *n* = 5 mice/group. (D) N17350 efficacy 72 h after a single intra-tumoral injection across various xenograft models; n = 5–7 mice/group. (E) Cancer and non-cancer cells were isolated from OvCa patients, treated with N17350 (500 nM), doxorubicin (10 μM), or oxaliplatin (100 μM) for 24 h, and cell viability was assessed; *n* = 2–3/patient, *n* = 31–39 patients/group. (F) Representative N17350 killing curve (left) and eXEC50 values (right) for cancer cells and CD45^+^ immune cells isolated from the same tumor of OvCa patients (*n* = 3/dose across patients). EC50 values for immune cells were assigned as >10,000 nM, as the maximal tested dose failed to achieve a response plateau, precluding accurate curve fitting. ∗*p* < 0.05, two-way ANOVA (C and E), Student’s t test: unpaired (D) and paired (F). Results are mean ± SEM. Arrows indicate N17350 treatment. All replicates are independent biological replicates.

To begin, we tested N17350’s ability to kill cancer cells with varying genetic profiles by assessing its potency (EC50) against a broad range of cancer cell lines and primary cancer cells isolated from ovarian cancer (OvCa) patients (Figure S2). N17350 effectively killed all cancer cells tested, demonstrating comparable efficacy across lung, breast, colon, ovarian, melanoma, and other cancer types (Figures 1A, 1B, and S3). It also killed lung cancer cells with distinct KRAS mutations (G12C, G12D, G12S, and G12V) and was effective against primary cancer cells isolated from 16 high-grade serous ovarian cancer (HGSOC) patients (Figures 1A, 1B, and S3), a heterogeneous cancer lacking specific driver mutations.^12^

Next, we tested N17350 in 12 genetically diverse syngeneic and xenograft models of lung cancer (NCI-H2122, NCI-H358, NCI-H1373, NCI-H441, and A549), colon cancer (CT26, MC38, HCT116, and HT29), breast cancer (4T1), prostate cancer (PC3), and esophageal cancer (KYSE-410). N17350 rapidly regressed tumors of varying size, genetics, and immunotype (hot: CT26, warm: MC38, cold: 4T1, xenografts) with similar potency (Figures 1C and 1D). N17350 also demonstrated improved efficacy compared to carboplatin, a standard-of-care chemotherapy, across many models or to KRAS-targeted therapies in lung cancer models (Figures 1C and S4). These results show that N17350 consistently provides effective tumor regression across genetically diverse tumors, both within and across tumor types.

We also assessed whether N17350 could selectively kill cancer cells while preserving immune cells. In a study using OvCa-patient-derived cells (Figure S2), N17350 selectively killed cancer cells while preserving CD45^+^ immune cells and fibroblasts isolated from the same patient’s tumor, omental tissue, intraperitoneal fluid, or blood, whereas oxaliplatin and doxorubicin indiscriminately killed all cell types (Figure 1E). Furthermore, N17350 exhibited an estimated >100-fold therapeutic window when tested on cancer cells and CD45^+^ immune cells from the same OvCa tumor (Figures 1F and S3A–S3C), demonstrating its selective killing property in patient samples. Selective killing was also observed with all cancer and non-cancer cell lines tested, and as expected,^13^ this effect was not due to higher N17350 uptake by cancer cells compared to non-cancer cells (Figures S3G and S3H).

### N17350-mediated tumor regression activates anti-tumor immunity

The mechanism by which cytotoxic agents induce cancer cell death is also critical for subsequent immune activation. Immunogenic cell death (ICD) is one such mechanism, promoting T cell activation by releasing tumor-associated antigens and damage-associated molecular patterns.^14^ To determine if N17350 induces ICD, we treated primary OvCa cells with N17350 or chemotherapeutic agents known for their ICD properties^8^^,^^15^^,^^16^^,^^17^ and measured key ICD markers such as cell surface calreticulin (CALR), high-mobility group box 1 protein (HMGB1), annexin A1 (ANXA1), and ATP release.

**Figure 2:**
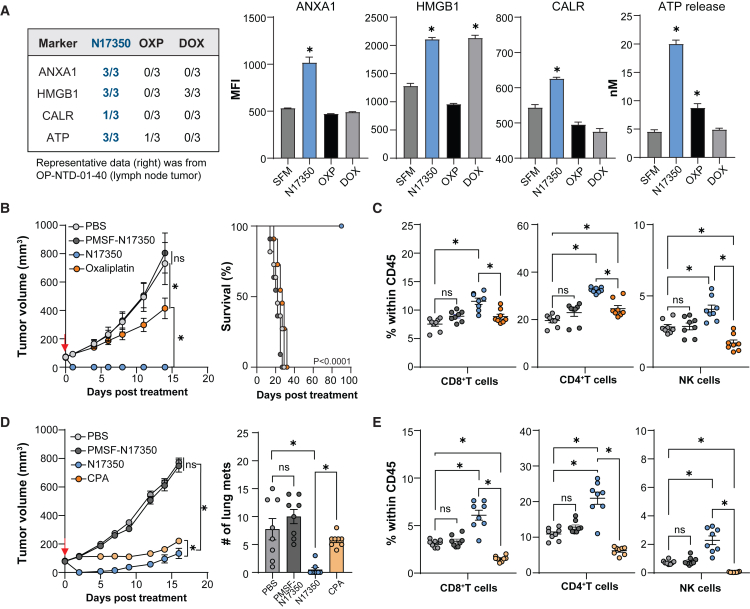
N17350 kills cancer cells via ICD and induces systemic immune cells (A) Cancer cells were isolated from primary tumors of OvCa patients, treated with N17350 (500 nM), oxaliplatin (OXP, 100 μM), or doxorubicin (DOX, 10 μM) for 24 h and ICD markers including extracellular ATP, and cell surface ANXA1, CALR, and HMGB1 were quantified (*n* = 3/patient, 3 patients). Representative data for OP-NTD-01-40 patient’s lymph node tumor (right) and summarized data across all patients (left) are shown (number of patient samples elevated/tested). (B and C) CT26-tumor-bearing mice were treated with N17350 or PMSF-N17350 (400 μg/100 mm^3^, intra-tumoral, red arrow), oxaliplatin (6 mg/kg, intraperitoneal, days 0 and 2), or vehicle, and effects on tumor growth (B) and systemic immune cells (C, day 12) were quantified (*n* = 8 mice/group). (D and E) 4T1-tumor-bearing mice were treated with N17350 or PMSF-N17350 (400 μg/100 mm^3^, intra-tumoral), cyclophosphamide (CPA, 100 mg/kg, intraperitoneal, days 0 and 2), or vehicle (PBS), and primary tumor growth, lung metastases (D, day 10), and systemic immune cells (E, day 12) were quantified (*n* = 8 mice/group). ∗*p* < 0.05, two-way ANOVA (B–E), Student’s t test: unpaired (A). Results are mean ± SEM. Arrows indicate N17350 treatment. All replicates are independent biological replicates.

N17350 increased ICD markers across all OvCa-patient-derived cancer cells tested (Figure 2A). Additionally, N17350 triggered ICD markers in both human and murine cancer cell lines (Figure S5), highlighting its broad ability to activate this immune-stimulating cell death process.

Based on N17350’s ability to induce ICD and preserve immune cells, we hypothesized that it could stimulate anti-tumor immunity *in vivo*, as previously reported for ELANE and PPE.^13^ We tested this in both the immunologically “hot” CT26 colon cancer model and the immunologically “cold” 4T1 model of metastatic breast cancer.^18^ Tumor-bearing mice (∼100 mm^3^) were treated with a single dose of N17350 (intra-tumoral) or various chemotherapies (two doses, intraperitoneal). N17350 rapidly regressed tumors and increased levels of CD8^+^ T cells, CD4^+^ T cells, and natural killer (NK) cells in the blood, and all these effects required enzymatic activity as they were not observed with catalytically inactive PMSF-N17350 (Figures 2B–2E). In contrast, oxaliplatin (CT26 model) and cyclophosphamide (4T1 model) only slowed tumor growth and had minimal or negative effects on systemic immune cells (Figures 2B–2E).

**Figure 3:**
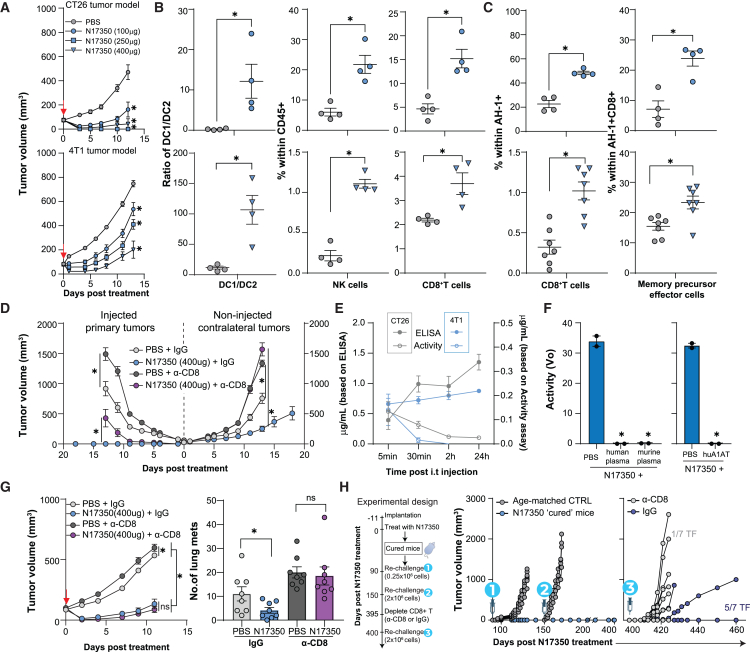
N17350-mediated tumor regression induces anti-tumor immunity (A–C) N17350 was injected into tumors in the CT26 and 4T1 (100–400 μg/100 mm^3^) models. Tumor growth responses (A); natural killer (NK) immune cell, CD8^+^ T cell, and dendritic cell profiles within live CD45^+^ in tumors (B); and AH-1^+^-antigen-specific CD8^+^ T cells and memory precursor effector CD8^+^ T cells in blood (C) 12 days post-treatment; *n* = 4–7 mice/group. (D) Effects of depleting CD8^+^ T cells on N17350 efficacy in primary and contralateral tumors in a dual flank CT26 model. (E) N17350 enzymatic activity and protein levels in plasma following intra-tumoral injection (100 μg/100 mm^3^) into CT26 or 4T1 tumors. (F) N17350 (400 nM) was incubated in PBS, human or murine plasma, or human A1AT (2 μM), and enzymatic activity was quantified. (G) Effects of depleting CD8^+^ T cells on N17350 efficacy in 4T1 primary tumor (left) and lung metastases (right); *n* = 8 mice/group. (H) Experimental design (left). CT26-tumor-bearing mice “cured” with a single dose of N17350 (A) were rechallenged with CT26 cells 90 days (0.25 × 10^6^ cells) and 150 days (2 × 10^6^ cells) post-N17350 treatment (middle) and 400 days (2 × 10^6^ cells) following treatment with anti-IgG and anti-CD8 antibodies (right). ∗*p* < 0.05, two-way ANOVA (A, D, and G), Student’s t test (B, C, and F). Results are mean ± SEM. Arrows indicate N17350 treatment. All replicates are independent biological replicates.

To further investigate the immune response triggered by N17350, we treated CT26 and 4T1 tumors with a single dose of N17350 and analyzed immune cell populations in the tumor and blood 12 days after treatment (Figures S6A–S6C). N17350 increased the DC1/DC2 ratio, NK cells, and CD8^+^ T cells in the tumor in both models (Figures 3A, 3B, S6D, and S6E). Additionally, N17350 induced systemic immune responses, including an increase in tumor-antigen-specific AH-1+ CD8^+^ T cells, which were enriched in memory precursor effector subsets (Figures 3C, S6D, and S6E). These results demonstrate that N17350-mediated tumor regression stimulates both local and systemic immune responses, suggesting the potential of generating a second wave of efficacy driven by anti-tumor immunity. This hypothesis was further explored using four experimental approaches.

First, we investigated whether N17350 could induce abscopal effects in a dual flank CT26 model. Treatment with N17350 led to rapid regression of the primary (injected) tumor and reduced tumor growth at the secondary site (Figure 3D). Importantly, this abscopal effect was not due to N17350 “spillover” from the primary site, as the small amount of N17350 detected systemically after intra-tumoral injection was rapidly inactivated by serine protease inhibitors such as alpha-1-anti-trypsin (A1AT), a property maintained in human plasma (Figures 3E, 3F, and S14). Depleting CD8^+^ T cells abolished the abscopal effect and diminished primary tumor control, without affecting the initial N17350-induced primary tumor regression (Figures 3D and S6F). Therefore, while CD8^+^ T cells are not required for N17350-mediated primary tumor regression, they are crucial for sustaining the response through adaptive immunity.

Second, we studied the impact of N17350 on spontaneous lung metastasis in the 4T1 model. Treatment of the primary tumor with N17350 reduced lung metastases, an effect that was eliminated by CD8^+^ T cell depletion (Figure 3G). This finding is particularly noteworthy in the 4T1 model, which is considered immunologically “cold” and resistant to engaging adaptive immunity.^19^^,^^20^ In contrast, treating 4T1 mice with cyclophosphamide (CPA) did not impact lung metastases, despite showing comparable effects on the primary tumor (see Figure 2D). This suggests that cyclophosphamide is neither able to directly kill metastatic cancer cells nor induce anti-tumor immunity (through primary tumor killing) to lessen metastasis in this model, perhaps due to its observed cytotoxic effects on CD8^+^ T cells (see Figure 2E).

Third, we evaluated immune memory by rechallenging CT26 “cured” mice with CT26 cells at two time points: 90 days (0.25 × 10^6^ cells) and 150 days (2 × 10^6^ cells) after N17350 treatment. All cured mice resisted rechallenges, while age-matched controls developed tumors (Figure 3H), and this immune memory was specific since CT26-cured mice readily developed 4T1 tumors (Figures S7A–S7C).

To explore the role of CD8^+^ T cells in immune memory, we depleted CD8^+^ T cells in half of the cured mice, while the other half received an immunoglobulin G (IgG) control antibody. When we rechallenged the cured mice a third time (2 × 10^6^ cells) 400 days post-treatment, CD8^+^ T cell depletion led to tumor growth in most mice (1/7 tumor-free), whereas the IgG control group maintained immune memory, with five of seven mice remaining tumor-free, even at advanced age (Figure 3H). N17350 also induced a specific immune memory response in MC38-cured mice, which resisted rechallenge with MC38 cells but not B16F10 cells (Figures S7D–S7F).

Adaptive immunity could also be induced by injecting CT26 cells killed with oxaliplatin into naive mice as part of a standard ICD assay,^21^^,^^22^ (Figure S8) but it could not be triggered by treating CT26-tumor-bearing mice with this chemotherapeutic agent (see Figure 2C). Thus, while oxaliplatin is an established ICD inducer^23^^,^^24^ and our findings confirm this property, it fails to activate anti-tumor immunity *in vivo*, likely due to its toxicity to essential immune cells (see Figure 1E). These findings are consistent with the idea that selective cancer cell killing is a critical factor for enhancing the immune activation potential of cytotoxic agents.

**Figure 4:**
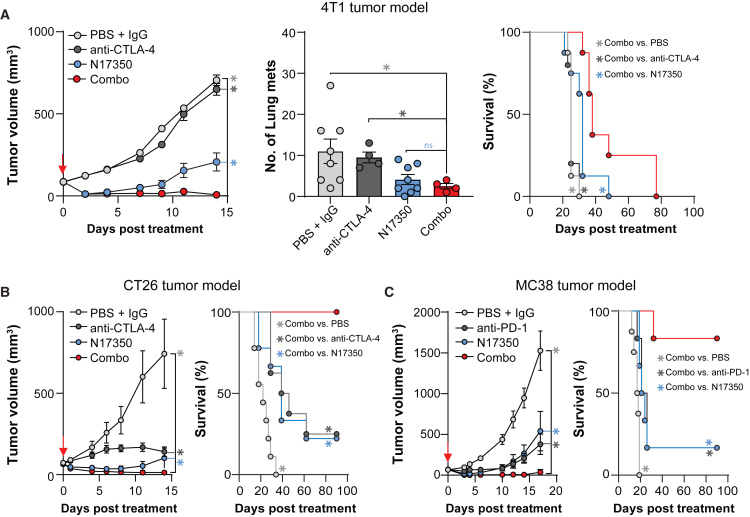
N17350 enables and/or improves checkpoint inhibitor efficacy (A) Effects of N17350 (400 μg/100 mm^3^, intra-tumoral, day 0) and anti-CTLA-4 (5 mg/kg; i.p.; days 0, 3, and 6), alone or in combination, in the 4T1 tumor model. Primary tumor growth (left), lung metastases (middle), and overall survival (right); *n* = 8 mice/group. (B) Effects of N17350 (100 μg/100 mm^3^, intra-tumoral, day 0) and anti-CTLA-4 (5 mg/kg; i.p.; days 0, 3, and 6), alone or in combination, in the CT26 model. Tumor growth (left) and overall survival (right); *n* = 8/group. (C) Effects of N17350 (250 μg/100 mm^3^, intra-tumoral, day 0) and anti-PD-1 (10 mg/kg; i.p.; days 0, 3, and 6), alone or in combination, in the MC38 model. Tumor growth (left) and overall survival (right); *n* = 6 mice/group. Note: a lower N17350 dose was used in the CT26 and MC38 models as higher doses eliminated tumors. ∗*p* < 0.05, two-way ANOVA; Mantel-Cox test (survival). Results are mean ± SEM. Arrows indicate N17350 treatment. All replicates are independent biological replicates.

Fourth, we tested whether N17350 could enhance the efficacy of CPIs in the 4T1 model, where anti-CTLA-4 treatment alone was ineffective (Figure 4A). A single dose of N17350 attenuated primary tumor growth and reduced lung metastases, while also enabling anti-CTLA-4 efficacy, leading to improved tumor control and survival (Figure 4A). N17350 also enhanced the efficacy of anti-CTLA-4 in the “hot” CT26 model (Figure 4B) and boosted anti-PD-1 effectiveness in the “warm” MC38 model of colon cancer (Figure 4C). In contrast, combining CPIs with oxaliplatin resulted in minimal improvement with anti-PD-1 and no improvement with anti-CTLA-4 in the CT26 model (Figure S9).

Altogether, these results underscore N17350’s potential to activate anti-tumor immunity, combining immediate local therapeutic effects with abscopal effects, long-term immune memory, and extending the efficacy of CPIs in treating challenging cancer types.

### N17350 maintains potency following repeated dosing

**Figure 5:**
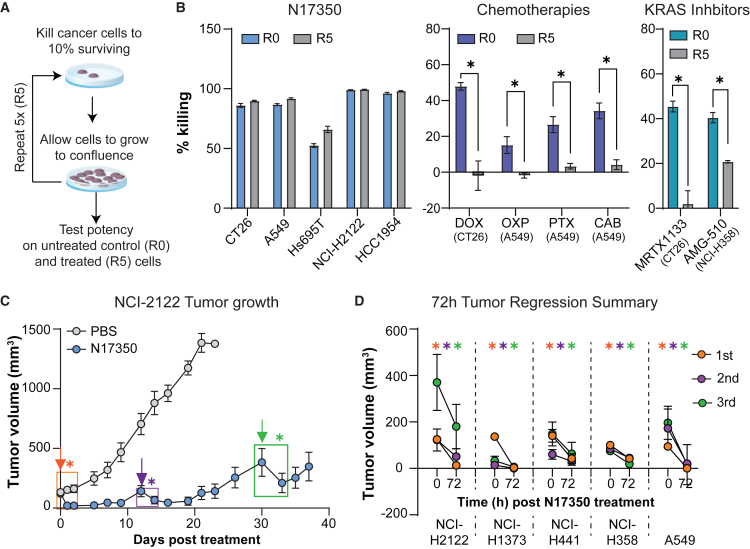
N17350 maintains potency following repeated dosing (A) Various cancer cell lines were killed five times (10% survival, regrow, and repeat) with different cytotoxic agents (R5) or serially passaged five times as a control (R0). (B) Effect of N17350 (500 nM, 24 h), doxorubicin (DOX, 16.7 μM, 72 h), oxaliplatin (OXP; 50 μM, 72 h), paclitaxel (PTX; 1.5 μM, 72 h), carboplatin (CAB; 200 μM, 72 h), MRTX1133 (3.1 nM, 72 h), or AMG-510 (6.3 nM, 72 h) on the viability of R0 and R5 cancer cells; *n* = 3–6/group. (C and D) Efficacy (72 h) of three successive N17350 treatments (400 μg/100 mm^3^, intra-tumoral) across five human lung cancer models. Results are displayed as tumor volumes pre- and 72 h post-N17350 treatment; *n* = 5 mice/group. (C) Representative data in the NCI-H2122 cancer model; arrows indicate treatment with N17350.(D) Summary of results across all models. ∗*p* < 0.05, Student’s t test: unpaired (B) and paired (C and D). Results are mean ± SEM. Arrows indicate N17350 treatment. All replicates are independent biological replicates.

The development of resistance is a major hurdle in the effectiveness of many cytotoxic therapies. To assess whether cancer cells could acquire resistance to N17350, we treated cancer cells with N17350 to achieve ∼90% killing. Cells were also treated with a range of chemotherapies (doxorubicin, oxaliplatin, paclitaxel, and carboplatin) or molecular therapies (KRAS inhibitors: AMG-510 and MRTX1133) as positive controls.^25^^,^^26^^,^^27^ Afterward, surviving cells were allowed to regrow to confluence, and this cycle was repeated five times. We then compared the potency of these cytotoxic agents on both the treated (R5) cells, which had undergone five rounds of killing, and the serially passaged untreated control (R0) cells (Figure 5A).

While five rounds of treatment were sufficient to induce resistance to all chemotherapies and KRAS inhibitors tested, this was not the case for N17350 (Figure 5B). N17350 maintained its efficacy after five successive killing cycles, indicating that cancer cells fail to acquire resistance under these conditions.

We further investigated whether resistance to N17350 could develop *in vivo* by re-analyzing data from five human lung cancer models treated with N17350 (see Figure S4). A single injection of N17350 did not result in complete tumor ablation in these xenograft models, perhaps due to the lack of a functional adaptive immune system, which plays a role in maintaining long-term control of the primary (injected) tumor as shown in the CT26 model (see Figures 3D–3H). This caveat allowed us to re-administer N17350 into the same tumor once it regrew and determine whether resistance emerged over time. Efficacy was assessed by quantifying effect on tumor size 72 h after each of three successive N17350 treatments per tumor. N17350 maintained its potency after each treatment in all five tumor models (Figures 5C and 5D), demonstrating that resistance to N17350 does not develop following repetitive dosing *in vivo*.

### N17350 effectively treats therapy-resistant tumors

Patients who acquire resistance to one drug can exhibit cross-resistance to others, both within and across drug classes, narrowing treatment options and limiting therapeutic efficacy.^28^ Given the frequent use of chemotherapies and immunotherapies in the clinic, we investigated whether resistance acquired to these agents affects N17350 efficacy.

**Figure 6:**
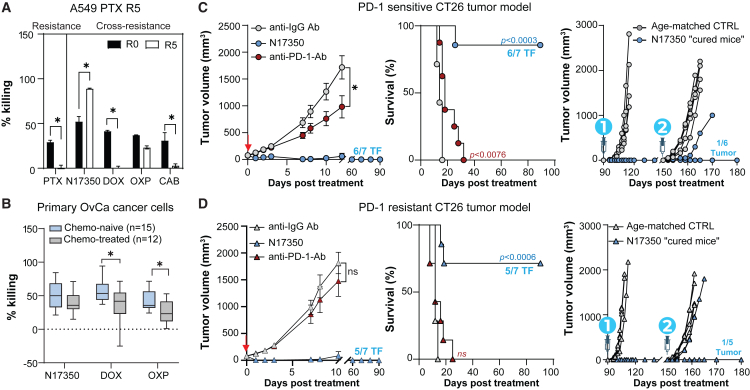
N17350 effectively treats therapy-resistant tumors (A) Previously established control (R0) and paclitaxel-resistant (R5) A549 cells (see Figure 5) were treated with N17350 (500 nM, 24 h), paclitaxel (PTX; 0.6 μM, 72 h), doxorubicin (DOX; 1.9 μM, 72 h), oxaliplatin (OXP; 33.3 μM, 72 h), or carboplatin (CAB; 66.7 μM, 72 h), and cell viability was assessed; *n* = 3/group. (B) Effects of N17350, DOX, and OXP on the viability of primary cancer cells from OvCa patients. Data presented in Figure 1E were re-analyzed after stratifying OvCa patients based on chemotherapy treatment history. (C and D) Effects of N17350 (400 μg/100 mm^3^, intra-tumoral), anti-PD-1, or IgG control antibodies (10 mg/kg; i.p.; days 0, 3, 6, and 9) on initial tumor growth (left), survival (middle), and tumor growth in “cured mice” following rechallenge with CT26 cells 90 days (0.25 × 10^6^ cells) and 150 days (2 × 10^6^ cells) post-treatment with N17350 (right) in PD-1-sensitive (C) and PD-1-resistant (D) mice; *n* = 5–7 mice/group. ∗*p* < 0.05, two-way ANOVA (C and D); Student’s t test (A and B); Mantel-Cox test (survival). Results are mean ± SEM. Arrows indicate N17350 treatment. All replicates are independent biological replicates.

To assess the effect of resistance acquired to chemotherapies on N17350-mediated cell killing, we treated paclitaxel-resistant cells (see Figure 5B) with N17350 or other chemotherapeutic agents. While doxorubicin and carboplatin showed reduced efficacy in killing cancer cells with acquired resistance to paclitaxel, N17350 retained full cytotoxic activity (Figure 6A). Similar findings were observed with other cancer cell lines that acquired resistance to chemotherapies and KRAS inhibitors (Figure S10). Additionally, N17350 was equally effective at killing cancer cells from both chemotherapy-experienced and chemotherapy-naïve patients, whereas doxorubicin and oxaliplatin were less effective in chemotherapy-experienced patients (Figure 6B).

We also examined how immunotherapy resistance affects N17350’s efficacy. CT26 tumors, initially sensitive to CPIs, were serially passaged in mice treated with an anti-PD-1 antibody to create a PD-1-resistant model^29^ intended to provide insights into CPI-progressing patients in the clinic. Whereas PD-1-resistant tumors showed resistance to anti-PD-1 treatment, N17350’s ability to regress tumors, extend survival, and generate immune memory remained unchanged (Figures 6C and 6D). These findings suggest that N17350 might elicit robust anti-tumor effects across a wide range of patient treatment histories, as commonly encountered in early-phase clinical trials.

### N17350 targets the ELANE pathway to treat patient-derived tumors

Our studies highlight N17350’s potent, broad-spectrum anti-cancer activity, marked by selective cancer cell killing and stimulation of anti-tumor immunity. These characteristics align closely with the ELANE pathway, which induces genotype-independent cytotoxicity in cancer cells while sparing immune cells. Hallmarks of ELANE pathway activation include proteolytic cleavage of CD95 to release its death domain, cytosolic translocation of histone H1 isoforms, induction of DNA damage, mitochondrial dysfunction, and activation of downstream apoptotic effectors.^13^^,^^30^^,^^31^

**Figure 7:**
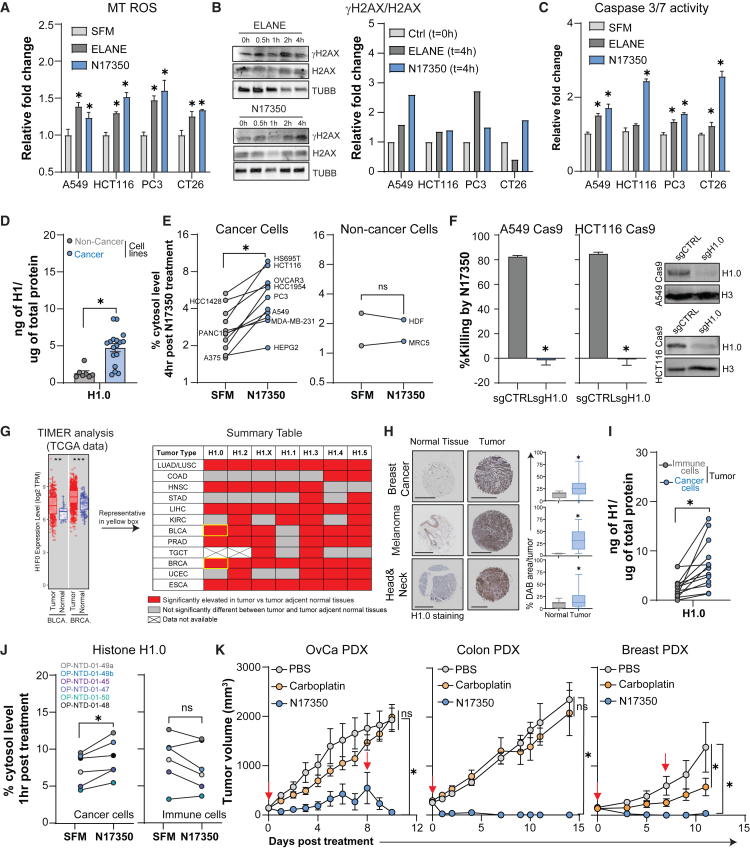
N17350 targets the ELANE pathway to treat patient-derived tumors (A–C) Cancer cells were treated with N17350 or ELANE, and hallmarks of the ELANE pathway were assessed. (A) CM-H2DCFDA fluorescence (mitochondrial [MT] ROS marker) post-treatment (500 nM, 30 min). Data were normalized to serum-free media (SFM) control (*n* = 3/group). (B) Western blot analysis of γH2AX/H2AX ratio (DNA damage marker) post-treatment (200 nM, 0–4 h). Representative immunoblots in A549 cells (left); 4-h analysis (right); (C) Caspase-3/7 activity (cell death effector) post-treatment (500 nM, 6 h). Data were normalized to SFM control (*n* = 3/group). (D) Histone H1.0 levels in cancer and non-cancer cell lines measured by ELISA and normalized to total protein. (E) Cytosolic translocation of histone H1.0 post-N17350 (200 nM, 4 h) or SFM treatment was quantified by flow cytometry and normalized to total H1.0. (F) Effect of H1.0 knockdown on N17350 (31.3 nM, 6 h) killing of A549 and HCT116 cells (left) and validation of knockdown efficiency at 72 h (right); *n* = 3/group. (G) TCGA analysis of histone H1 isoform levels in tumor tissue versus tumor-adjacent normal tissue across many solid tumor types. Tumors with significantly elevated histone H1 gene expression are shaded in red (*p* < 0.05, Wilcoxon test). (H) Tumor microarrays from melanoma, breast, and head and neck cancer patients were stained with anti-histone H1.0, using normal tissue as a control. Representative image (left) and quantification (right). Scale bars, 1 mm. (I) H1.0 levels in primary cancer cells and CD45^+^ immune cells isolated from the same tumor of OvCa patients. (J) Effect of N17350 (500 nM, 1 h) on histone H1.0 and H1.2 cytosolic translocation in primary cancer cells or CD45^+^ immune cells from tumors or intraperitoneal fluid of the same patient. (K) Effect of N17350 (400 μg/100 mm^3^, intra-tumoral) or carboplatin (100 mg/kg, i.p., days 0 and 7) on tumor growth in OvCa (left), colon cancer (middle), and breast cancer (right) patient-derived models; *n* = 5 mice/group. ∗*p* < 0.05, Student’s t test: unpaired (D, F, and H) and paired (E, I, and J), two-way ANOVA (A, C, and K). Results are mean ± SEM. Arrows indicate N17350 treatment. All replicates are independent biological replicates.

To determine whether N17350 engages this pathway, we evaluated its ability to recapitulate ELANE-mediated hallmarks in multiple cancer cell lines. N17350 induced all key features of the ELANE pathway, including γH2AX accumulation (indicative of DNA damage), CM-H2DCFDA fluorescence (mitochondrial ROS), and caspase-3/7 activity (cell death effector) (Figures 7A–7C and S11).

The cancer-selective nature of the ELANE pathway is mediated in part by histone H1 isoforms, which are elevated in various cancer cell lines^32^ and more readily translocate to the cytosol in cancer cells after ELANE treatment.^13^ We used several complementary approaches to assess the role of histone H1 isoforms in N17350-mediated cytotoxicity.

Expression profiling revealed significantly higher H1.0 and H1.2 protein levels in cancer cell lines compared to non-cancer cells (Figures 7D and S12A). Subcellular localization studies showed rapid cytosolic translocation of H1.0 and H1.2 in cancer cells, but not in non-cancer cells, after N17350 treatment (Figures 7E and S12B). CRISPR knockdown of H1.0 or H1.2 in A549 Cas9 and HCT116 Cas9 cancer cells substantially reduced N17350-induced cell death, consistent with prior findings for ELANE and PPE (Figures 7F and S12C).^13^ These results demonstrate that histone H1.0 and H1.2 contribute, in part, to the selective vulnerability of cancer cells to N17350.

To assess clinical relevance, we analyzed histone H1 isoform expression across tumor types using The Cancer Genome Atlas (TCGA) and TIMER. Histone H1 isoforms were significantly overexpressed in all solid tumors examined, compared to matched adjacent non-tumor tissues (Figure 7G). Immunostaining of tumor microarrays from melanoma, breast, and head and neck cancer patients confirmed elevated H1.0 and H1.2 protein levels in tumor versus non-tumor tissue (Figures 7H, S12D, and S13). Moreover, analysis of OvCa tumors revealed higher levels of H1.0 and H1.2 in cancer cells than in CD45^+^ immune cells from the same OvCa tumors (Figures 7I and S12E). Upon N17350 treatment, histone H1.0 and H1.2 translocated to the cytosol in cancer cells from OvCa patients but not in matched CD45^+^ immune cells from the same tumor (Figures 7J and S12F).

These findings highlight the elevated expression and dynamic localization of histone H1 isoforms in cancer cells, particularly in response to N17350 treatment, underscoring the broad potential of targeting the ELANE pathway across a wide range of tumor types. To investigate this possibility pre-clinically, we assessed N17350 efficacy in patient-derived models of ovarian, breast, and colon cancer. Consistent with data across 12 other pre-clinical models, N17350 produced prompt and potent tumor regression and outperformed carboplatin across all patient-derived models tested (Figure 7K).

## Discussion

Recent clinical studies have demonstrated improved outcomes in patients receiving various combinations of cytotoxic agents and immunotherapy,^4^^,^^5^^,^^33^^,^^34^ highlighting the complementary nature of these approaches and establishing a need for next-generation agents that combine the benefits of both therapies. In this study, we leveraged the ELANE pathway to develop N17350, a cytotoxic agent designed to activate anti-tumor immunity by selectively killing cancer cells. We thoroughly assessed N17350’s therapeutic properties and mechanisms using a comprehensive evaluation across 30 cancer cell lines, 15 tumor models, 45 patient samples, and 6 standard-of-care agents, employing experimental frameworks designed to mirror the complexity of clinical settings in which N17350 might first enter the clinic. Below, we discuss the underlying mechanistic and physiological processes driving this emerging therapeutic approach and contextualize it within current treatment strategies.

Cytotoxic agents have the potential to activate the immune system by triggering the release of tumor antigens and immune-stimulating molecules during immunogenic cell death. In principle, this potential hinges on their ability to (1) broadly kill cancer cells to overcome tumor heterogeneity, while (2) maintaining selectivity to preserve immune cells, which can then capitalize on the antigens released from dying cancer cells.

Achieving the right balance between maximizing tumor cell death and preserving immune cell function is a critical challenge in developing cytotoxic agents that can also effectively engage the immune system. This challenge is exemplified by our studies with oxaliplatin, which, while capable of killing many cancer types and inducing anti-tumor immunity in standard ICD assays, failed to activate anti-tumor immunity when administered therapeutically to tumor-bearing mice. Adaptive immune activation is also rarely observed with oxaliplatin or other chemotherapies in the clinic, due in part to their toxicity to immune cells.^33^^,^^35^

To address this issue, we developed N17350 to target the ELANE pathway, with histone H1 isoforms playing a key role in its selective action.^13^ Through an analysis of TCGA data; fresh tumor tissue from OvCa patients; tumor microarrays from breast, lung, and skin cancers; and cell lines representing a range of cancer types, we present strong evidence that histone H1.0 and H1.2 are elevated in many cancers and preferentially translocate out of the nucleus in cancer cells after treatment with N17350. These molecular findings were associated with (1) broad cancer killing, showing comparable efficacy across a wide range of genetically distinct cancer cell lines and primary cancer cells from OvCa patients, and (2) selective cancer killing, as indicated by an estimated >100-fold therapeutic index between the killing of cancer cells and CD45^+^ immune cells isolated from the same OvCa patient tumor.

Elevated histone H1 levels in cancer have been reported by other studies,^32^^,^^36^ and the importance of H1 translocation in the therapeutic efficacy of PPE and other cytotoxic agents has been demonstrated.^37^^,^^38^ Thus, histone H1 might serve as an attractive therapeutic target that is upregulated across many cancer types, perhaps due to its ability to support aberrant gene expression, promote uncontrolled cell proliferation, and maintain genomic stability^39^^,^^40^^,^^41^—functions essential for the survival and fitness of most, if not all, cancer cells. Moreover, histone H1 levels might serve as a biomarker for selecting or stratifying patients for clinical trial recruitment and could also predict the likelihood of treatment response, a readily testable hypothesis in forthcoming clinical trials of N17350.

Targeting the ELANE pathway confers several distinguishing therapeutic properties to N17350 that set it apart from currently available cytotoxic therapies. First, N17350 regresses tumors in a genotype-independent manner. We demonstrated this capability across 15 distinct preclinical models, spanning syngeneic, xenograft, and patient-derived models of breast, colon, esophageal, lung, ovarian, and prostate cancer. Its ability to overcome tumor heterogeneity is highlighted by both its broad action across tumor types and subtypes. This distinguishes N17350 from molecular therapies that target specific genotypes. For example, unlike KRAS G12C-targeted therapies such as MRTX1257 and AMG-510, N17350 showed robust efficacy across a variety of lung cancer models with variable KRAS mutations including, G12C, G12D, G12S, and G12V mutations.

Second, N17350-mediated cancer cell destruction triggers anti-tumor immunity. A single intra-tumoral injection of N17350 increased the DC1/DC2 ratio and boosted CD8^+^ T cells in treated tumors. It also enhanced the presence of antigen-specific AH1^+^ CD8^+^ T cells with memory characteristics in the bloodstream. Additionally, depleting CD8^+^ T cells reduced the durability of treatment responses and long-term immune memory in the CT26 model and eliminated the reduction of lung metastases in the 4T1 model. These results demonstrate that selectively targeting tumors with N17350 activates adaptive immunity, enhancing local efficacy and extending it to distant sites.

The ability to trigger anti-tumor immunity regardless of the tumor’s immunotype could support the combination of N17350 with CPIs, enhancing their effectiveness in difficult-to-treat tumor types (as shown in “cold” 4T1 tumors) and/or boosting their efficacy in tumors that respond to treatment (as shown in “warm” MC38 and “hot” CT26 tumors). Several clinical trials are currently testing CPIs in combination with chemotherapy or other targeted therapies across different tumor types. This approach shows promise,^42^ even though cytotoxic agents are generally not designed to activate anti-tumor immunity and seldom exhibit this effect when administered as a monotherapy.^43^ It will be interesting to explore whether this promise can be maximized by combining CPIs with the dual therapeutic action offered by N17350.

Third, N17350 is a first-in-class cytotoxic agent with an orthogonal mechanism of action that maintains potency following repetitive treatments and avoids cross-resistance to common cancer therapeutics. The cancer treatment landscape is intricate, with multiple lines and sequences of therapy that differ by tumor type and subtype. This complexity creates a critical need for therapies that can overcome and cut across cross-resistance mechanisms that emerge as front-line treatments fail—an issue that is especially prominent during early-phase clinical trials. Cross-resistance is evident in our studies with chemotherapy- or KRAS-inhibitor-resistant cancer cells, comparisons between chemotherapy-naïve and experienced OvCa patients, and may contribute to why second- and third-line chemotherapies are often less effective when frontline treatments fail in clinical settings.^44^^,^^45^ We present compelling evidence that resistance developed to chemotherapies, KRAS inhibitors, or CPIs does not impact N17350 efficacy in pre-clinical models, thereby supporting its testing in phase I clinical trials involving heavily pre-treated patients.

Fourth, intra-tumorally administered N17350 demonstrates a favorable pharmacokinetic profile, offering both local activity to drive efficacy and systemic inertness to promote safety. This is evident in pre-clinical models, where we show that N17350 escape from the tumor results in its catalytic inactivation in the bloodstream, mediated by A1AT (Figure S14) and other serine protease inhibitors, which eliminate enzymatic activity in human plasma. By harnessing the body’s own serine protease mechanisms, N17350 achieves this beneficial pharmacokinetic profile without the need for additional targeting or masking strategies that might restrict its implementation.

As with all intra-tumoral therapeutics, N17350 must overcome key challenges to achieve clinical efficacy, including sufficient tumor penetration, prolonged intra-tumoral residence, and the capacity to elicit systemic anti-tumor immune responses from a localized injection. In preclinical models, N17350 demonstrated robust efficacy across a wide range of tumor sizes and models, suggesting effective tumor distribution. Moreover, pharmacokinetic studies revealed stable tumor retention of N17350 for at least 48 h post-injection (Figure S15), supporting a sufficient exposure window for selective cancer cell killing and initiation of anti-tumor immunity. Importantly, N17350 also elicited strong immune-activating effects across multiple tumor models, including those with distinct immunotypes, highlighting its potential to induce systemic anti-tumor immunity. In these settings, N17350 not only suppressed distal, non-injected tumors but also promoted durable immune memory and outperformed systemically administered cyclophosphamide in controlling metastatic disease.

Nonetheless, translating these effects into human patients, who present with larger, more heterogeneous, and immunologically complex tumors, will inevitably magnify these challenges. The success of N17350 in first-in-human trials will hinge on its ability to replicate its preclinical performance in the context of clinical tumor biology, immune variability, and mode of delivery.

Given the striking efficacy of N17350, both at the injection site and in distal tumors, it seems ideally suited for testing in clinical settings where direct intra-tumoral injections are most practical. The initial translation to patients with treatment-refractory conditions (such as squamous cell carcinomas of the head and neck) or those with various cutaneous cancers (e.g., melanoma, Merkel cell carcinoma, and squamous/basal cell carcinomas) will provide a straightforward approach for drug delivery, as well as pre- and post-treatment tumor sampling for biomarker analysis. Importantly, these patients will have diverse tumor genetics and immune profiles and will have undergone various prior treatments, including radiotherapy, chemotherapy, immunotherapy, and targeted therapies. These clinical scenarios will offer an opportunity to thoroughly examine and validate the preclinical findings outlined in this report.

Beyond superficial tumors, N17350’s clinical utility could extend to deep-seated visceral malignancies with advanced image-guided delivery platforms, enabling precise intra-tumoral delivery to anatomically challenging sites. Several of these platforms are currently in late-stage clinical trials, underscoring growing clinical adoption of this strategy. Parallel development of NEU-002, a modified elastase engineered to evade A1AT inhibition while retaining restricted catalytic activity in the bloodstream, offers a complementary approach for targeting visceral lesions via systemic delivery.^46^^,^^47^

Altogether, our studies demonstrate that N17350 produces strong therapeutic effects across tumors of distinct genotypes, immunotypes, and treatment history. Its therapeutic efficacy combines prompt tumor regression with chronic adaptive immune activation—properties that are enabled by its ability to target the ELANE pathway to broadly kill cancer cells via ICD, with a wide therapeutic index to preserve critical immune cells. These studies differentiate N17350 from other cytotoxic agents and provide strong rationale for testing in first-in-human clinical trials both as a monotherapy and in combination with CPIs.

### Limitations of the study

(1) Due to reagent availability and feasibility, studies of histone H1 levels and translocation were primarily restricted to the H1.0 and H1.2 isoforms. Additional studies are required to ascertain the expression and dynamics of other histone H1 isoforms.
(2) CD8^+^ T cell depletion failed to restore tumor growth in all CT26-“cured mice.” This observation suggests that additional immune cell types may contribute to immune memory generated by N17350 treatment. N17350-mediated tumor regression induced other immune cell populations; additional experiments are needed to ascertain their role in immune memory.
(3) While N17350 displays a wide therapeutic index (an estimated >100-fold) between the killing of cancer and non-cancer cells, extrapolating this result beyond the cell types tested in this paper requires experimental validation.

## Resource availability

### Lead contact

Requests for further information, resources, and reagents should be directed to and will be fulfilled by the lead contact, Lev Becker (lbecker@onchillespharma.com).

### Materials availability

This study did not generate new, unique reagents.

### Data and code availability

This study does not report original code or datasets. All analysis code and algorithms are described in the STAR Methods section. Additional information required to reanalyze the data is available from the lead contact upon request.

## Acknowledgments

This work was supported by private funds from Onchilles Pharma Inc. We thank Peter Haberz for acquisition of reagents, equipment, and contract management; Sonia Feau for assistance with the IRB and patient sample procurement; Christine Lee, Nicole Grigatis, Asna Khalid, and Hannah Liu for their technical assistance; and Carmen White and Roxanne Lix for their financial management.

## Author contributions

Conceptualization, L.B., C.C., R.G., and C.T.; methodology, L.B., C.C., and R.G.; formal analysis, L.B., C.C., and R.G.; investigation, C.C., R.G., M.F., and N.M.; writing—original draft, L.B. and C.C.; manuscript editing, R.G., C.C., M.F., N.M., A.B., A.A., K.H., C.T., and L.B.; funding acquisition, C.T. and L.B.; supervision, L.B.

## Declaration of interests

This research was performed at Onchilles Pharma Inc., a privately held biotechnology company in San Diego, CA, USA. L.B. is a co-founder, board member, chief scientific officer, and stockholder of Onchilles Pharma Inc. C.T. is a co-founder, board member, chief executive officer, and stockholder of Onchilles Pharma Inc. C.C., R.G., M.F., and N.M. are employees of Onchilles Pharma Inc. with stock options. A.A. is a paid consultant for Onchilles Pharma Inc. with stock options. A.B. is an investor in Onchilles Pharma Inc. K.H. declares advisory board/steering committee membership with Adlai Nortye, ALX, AstraZeneca, Aveo Pharmaceuticals, BeiGene Ltd., Bicara Therapeutics, BMS, Boehringer Ingelheim, Calliditas Therapeutics, Codiak BioSciences, Exelixis, Flamingo Therapeutics, Genmab, Gilead Sciences, GSK, Inovio Pharmaceuticals, ISA Pharmaceuticals, Johnson & Johnson, Merck Serono, Merus, MSD, Onchilles Pharma Inc., PDS Biotechnology, Pfizer, QBiotics, Replimune, and Rgenta Therapeutics (honoraria/fees to institution) and has received research funding from AstraZeneca, Boehringer Ingelheim, and Replimune (fees to institution). These studies are associated with the following patents: PCT/US2021/046467, Methods and Compositions for Treating Cancer; PCT/US2021/046453, Modified Porcine Pancreatic Elastase Proteins; and PCT/IB2024/060843, Histone H1 Levels as Biomarkers for Cancer Therapy.

## STAR★Methods

### Key resources table

**Table:** 

REAGENT or RESOURCE	SOURCE	IDENTIFIER
**Antibodies**		

Anti-CTLA-4	BioXCell	Cat#BP0131 Lot#755622A1; RRID: AB_10950184
Anti-PD-1	BioXCell	Cat#BP0146 Lot#842922M2B; RRID: AB_10949053
Anti-CD8	BioXCell	Cat#BP0061 Lot#86823M1; RRID: AB_1125541
Rat IgG2a isotype	BioXCell	Cat#BP0089 Lot#815021S1; RRID: AB_1107769
Rat IgG2b isotype	BioXCell	Cat#BP0090 Lot#767921D1; RRID: AB_1107780
Syrian Hamster IgG	BioXCell	Cat#BP0087 Lot#760821J1; RRID: AB_1107782
Anti-mouse CD8 antibody	BioLegend	Cat#100750 Lot#B369775; RRID: AB_11218801
Anti-mouse CD127 antibody	BioLegend	Cat# 135035 Lot#B384765; RRID:AB_2564577
Anti-mouse NKP46 antibody	BioLegend	Cat#137612 Lot#B353454; RRID: AB_10915472
Anti-mouse KLRG1 antibody	BioLegend	Cat#138409 Lot#B364525; RRID: AB_10643582
Anti-mouse PD-1 antibody	BioLegend	Cat#135216 Lot#B355884; RRID: AB_10689635
Anti-mouse CD3 antibody	BioLegend	Cat#100271 Lot#B359123; RRID: AB_2876394
Anti-mouse CD45 antibody	BioLegend	Cat#103144 Lot#1342647; RRID:AB_2563458
Anti-mouse CD4 antibody	BioLegend	Cat#100567 Lot#B314140; RRID: AB_2629699
Anti-mouse CD62L antibody	BioLegend	Cat#104412 Lot#B355945; RRID: AB_313098
Anti-mouse CD44 antibody	BioLegend	Cat#103047 Lot#B391672; RRID: AB_2562451
L/D Aqua	Invitrogen	Cat#L34957 Lot#2400851
Anti-mouse PD-L1 antibody	BioLegend	Cat# 124331 Lot#B330768; RRID: AB_2629659
Anti-mouse CD54 antibody	BioLegend	Cat#116143 Lot#B382455; RRID:AB_2876429
Anti-mouse CD11b antibody	BioLegend	Cat#101259 Lot#B350045; RRID: AB_11125575
Anti-mouse CD86 antibody	BioLegend	Cat#105037 Lot#B339994; RRID:AB_11204429
Anti-mouse BST2 antibody	BioLegend	Cat#127108 Lot#B302820; RRID: AB_2259316
Anti-mouse Ly-6G antibody	BioLegend	Cat#127615 Lot#B357443; RRID:AB_1877271
Anti-mouse Ly-6C antibody	BioLegend	Cat#128005 Lot#B336286; RRID:AB_1186134
CD206 antibody	BioLegend	Cat#141720 Lot#B371538; RRID: AB_2562247
Anti-human/mouse CD11c antibody	BioLegend	Cat#117307 Lot#B361852; RRID:AB_313776
Anti-mouse B220 antibody	BioLegend	Cat#103280 Lot#B344991; RRID: AB_2876408
Anti-mouse CD45 antibody	BioLegend	Cat#103144 Lot#B335041; RRID:AB_2563458
Anti-mouseI-A/I-E antibody	BioLegend	Cat#107652 Lot#B362982; RRID: AB_2616728
Anti-mouse CLEC9A antibody	BioLegend	Cat#143506 Lot#B345904; RRID: AB_2566379
Anti-mouse CD25 antibody	BioLegend	Cat#102038 Lot#B359383; RRID: AB_11125760
Anti-mouse Tim-3 antibody	BioLegend	Cat#119721 Lot#B340648; RRID: AB_2616907
Anti-mouse LAG-3 antibody	BioLegend	Cat#125212 Lot#B290991; RRID: AB_2561516
Anti-mouse KLRG1 antibody	BioLegend	Cat#138409 Lot#B364525; RRID: AB_10643582
Anti-mouse Foxp3 antibody	BioLegend	Cat#320008 Lot#B346122; RRID: AB_492980
Anti-mouse CD69 antibody	BioLegend	Cat#104513 Lot#B361544; RRID:AB_492843
Histone H3 Antibody	Cell Signaling Technology	Cat#9715L Lot#23
Alpha Tubulin	Cell Signaling Technology	Cat#2144S Lot#7
Anti-phospho-Histone H2A.X (Ser13) clone JBW301	Milipore-Sigma	Cat#05-636-25UG Lot#4266481
Histone H2A.X	Cell Signaling Technology	Cat#2595S Lot#11
Calreticulin m/h	Abcam	Cat# ab210431; Lot# GR3456561-1
HMGB1 m/h	BioLegend	Cat# 651404; Lot# B341492
ANXA1 m/h	Abcam	Cat# ab225511; Lot# 1010238-1
Anti-H1.0	Sigma	Cat# HPA000843; Lot# 000053129
Anti-HIST1H1C (H1.2)	Sigma	Cat# HPA055907; Lot# 000054285
Anti-H1.0	Abcam	Cat# ab11079; Lot# GR3413078-7
Anti-H1.2	Proteintech	Cat# 19649-1-AP; Lot#00012929
Anti-H1.2	Abcam	Cat# ab17677; Lot# GR3460651-1
Anti-CELA1	LSBio	Cat#LS-C19084; Lot#121236
Anti-CELA1 HRP	LSBio	Cat#LS-C209658; Lot#66012
Anti-human CD45 antibody	BioLegend	Cat#304050 Lot#B396110; RRID: AB_2563465
Anti-human CD3 antibody	BD Pharmigen	Cat#560835 Lot#1064842; RRID: AB_2033956
Anti-human/mouse CD11b antibody	BD Pharmigen	Cat#562793 Lot#0230974; RRID: AB_2737798
Anti-human CD19 antibody	BD Pharmigen	Cat#561742 Lot#0111590; RRID: AB_398597
Anti-human CD4 antibody	BioLegend	Cat# 317434 Lot#B352290; RRID: AB_11150413
Anti-human CD8 antibody	BioLegend	Cat# 344714 Lot#B341619; RRID: AB_2044006
PE-*anti*-rabbit IgG	BioLegend	Cat# 406421 Lot#B411654
Streptavidin-HRP	R&D Systems	Cat# 893019 Lot#P366832
Goat anti-rabbit IgG (HRP)	Invitrogen	Cat# 31460

**Biological samples**		

Breast cancer tissue array with normal breast tissue; 5 cases/5 cores	BioCoreUSA	Cat# B-05Bre-1
Breast cancer with adjacent normal breast tissue array; 72 cases/72 cores	BioCoreUSA	Cat# B-0801Bre-3e
Breast tumor survey tissue array; 208 cases/208 cores	BioCoreUSA	Cat# BR20832
Multiple head and neck tumor with normal tissue array; 80 cases/80 cores	BioCoreUSA	Cat# XHN-802c
Head and neck cancer tissue array with normal tissue; 80 cases/80 cores	BioCoreUSA	Cat# B-80Han-3
Malignant melanoma tissue array with adjacent normal skin tissue; 48 cases/48 cores	BioCoreUSA	Cat# B-48Mel-1e

**Chemicals, peptides, and recombinant proteins**		

BME, Type 3 Cultrex	R&D systems	Cat#3632-005-02 Lot#1582359
Phenylmethylsulfonyl fluoride (PMSF)	Millpore Sigma	Cas# 329-98-6
Calcein-AM Viability Dye	ThermoFisher Scientific	Cat# C1430
CM-H2DCFA Dye	Millpore Sigma	Cat# C6827
AAPV substrates	Milipore Sigma	Cat#324740
Protease inhibitor cocktail	Millpore Sigma	Cat# P8340
AMG-510	MedChemExpress	Cat#229672-00-3 Lot#159450
MRTX1257	MedChemExpress	Cat#2206736-04-9 Lot#49910
MRTX1133	MedChemExpress	Cat#HY-134813 Lot#251117
Carboplatin	Sigma	Cat#PHR3417; Batch#MKCR3593
Cyclophosphamide	Sigma Aldrich	Cat#PHR1404
Corn Oil	MedChemExpress	Cat#Hy-y1888
Oxaliplatin	Sigma Aldrich	Cat#PHR1528; Source# _RAC5719
Paclitaxel	Invitrogen	Cat#P3456; Lot#3123565
Doxorubicin	Selleckchem	Cat# S1208; Lot#S120816
RPMI-1640 Medium	Gibco	Cat#11-875-05
RBC lysis Buffer	BioLegend	Cat#420301
Antibiotic-antimycotic	Gibco	Cat#15240062
Fetal Bovine Serum (FBS)	Gemini Bio-Products	Cat#10-082-147
DMEM Medium	Gibco	Cat#11965-092
Vascular Cell Basal Medium	ATCC	Cat# PCS-100-030
Endothelial Cell Growth Kit-BBE	ATCC	Cat# PCS-100-040
Fibroblast Basal Medium	ATCC	Cat# PCS-201-030
Fibroblast Growth Kit-Low serum	ATCC	Cat# PCS-201-040
Pen-Strep	Gibco	Cat#15140-122
Sodium Pyruvate	Gibco	Cat#11360-070
McCoy’s Medium	Gibco	Cat#16600-82
AIM-V Medium	Gibco	Cat#12055-091
Phosphate Buffered Saline (PBS pH 7.4 (1X)	Gibco	Cat#10010-023
TrypLE Express	Gibco	Cat#12605-028
DNAase I	Sigma Aldrich	Cat#D4513
Hyaluronidase from bovine testes	Sigma Aldrich	Cat#H3506
Collagenase Type IV	Gibco	Cat#17104-019
Recombinant Histone H1.0	Wuxi Biologics	Customized Wbp7387
Recombinant Histone H1.2	Wuxi Biologics	Customized Wbp7387
CD95-C	Wuxi Biologics	Cat#WBP7387H Lot#20210204
Recombinant N17350 (zymogen)	Wuxi Biologics	Customized Wbp7220A
Biotin-NHS-ester	Broadpharm	Cat# BP-22106
pHrodo Deep Red TFP ester	ThermoFisher Scientific	Cat# P35359
Alexa Fluor 680 NHS ester	ThermoFisher Scientific	Cat# A20008
DMSO	Sigma-Aldrich	Cat#D2650; Lot#RNBL1928
1-Step™ Ultra TMB-ELISA	ThermoFisher Scientific	Cat# 34029
Stop Solution [2N Sμlfuric Acid]	R&D Systems	Cat# DY994
Sodium carbonate	Sigma-Aldrich	Cat# 223530-500G
Sodium bicarbonate	Sigma-Aldrich	Cat# S5761-500G
TWEEN 20	Sigma-Aldrich	Cat# P7949-500mL
Human alpha-1-*anti*-trypsin (A1AT)	SCRIPPS laboratories	Cat# 90082-0001
Bovine Serum Albumin/BSA	Sigma-Aldrich	Cat# A9647-100G
One-Step Blue Protein Gel Stain 1X	Biotium	Cat:21003
Novex Tris Glycine SDS Running Buffer 10x	ThermoFisher Scientific	Cat#LC26754
SuperSignal ELISA Femto Maximum Sensitivity Substrate	ThermoFisher Scientific	Cat# 37075

**Critical commercial assays**		

Zeba spin desalting columns 7K MWCO	ThermoFisher Scientific	Cat# 89890
CD45 microbeads	Miltenyi Biotec	Cat# 130-045-801
EPCAM microbeads	Miltenyi Biotec	Cat# 130-061-101
Fc blocker	Miltenyi Biotec	Cat# 130-059-901
PD-10 desalting column	GE Healthcare Life	Cat# 17-0851-01
eBioscience™ Foxp3/Transcription factor fixation/permeabilization concentrate and diluent	ThermoFisher Scientific	Cat# 00-5521-00
Flow staining buffer	BD Biosciences	Cat# 554657
Amicon Ultra-15 Centrifugal Filter Units	Millpore Sigma	Cat# UFC5100
0.22μm PES filter	Millpore Sigma	Cat# SLGPR33
BD Cytofix/Cytoperm fixation/permeabilization kit	BD biosciences	Cat# 554714
Aimcon Ultra-15 centrifugal filter unit 3kD MWCO	Millipore Sigma	Cat# UFC9003
Mag-Trypsin	Takara	Cat# 635646
Anti-mouse AH-1 Dextramer	Immudex	Cat#JG03294
Edit-R Predesign Human Human HIST1H1C	Dharmacon	Cat#SG-006630-01-0010
Edit-R Predesign Human Human H1F0	Dharmacon	Cat#SG-017209-01-0005
Edit-R synthetic sgRNA negative controls	Dharmacon	Cat#U-009501-01-05
DharmaFECT 4 Transfection Reagent	Dharmacon	Cat#T-2004-03
Ccaspase-Glo 3/7 Assay System	Promega	Cat# G8093
Mini-Protean TGX Stain-Free Gels 4–15%	Bio-rad Laboratories	Cat#4568086
Pierce BCA protein assay kits	ThermoFisher Scientific	Cat# 23227

**Experimental models: Cell lines**		

MC38	Kerafast (NIH)	ENH204-FP
CT26	ATCC	Cat# CRL-2638
4T1	ATCC	Cat# CRL-2539
B16F10	ATCC	Cat# CRL-6475
NCI-H2122	ATCC	Cat# CRL-5985
NCI-H1373	ATCC	Cat# CRL-5866
NCI-H441	ATCC	Cat# HTB-174
KYSE-410	Millipore-Sigma	Cat# 94072023
HCT116	ATCC	Cat# CCL-247
HT29	ATCC	Cat# HTB-38
A549	ATCC	Cat# CCL-185
SK-LU-1	ATCC	Cat# HTB-57
A375	ATCC	Cat# CRL-1619
HCT116	ATCC	Cat# CCL-247
HepG2	ATCC	Cat# HB-8065
PC3	ATCC	Cat# CRL-1435
FaDu	ATCC	Cat# HTB-43
A253	ATCC	Cat# HTB-41
E0771	ATCC	Cat# CRL-3461
MDA-MB-231	ATCC	Cat# HTB-26
OVCAR3	ATCC	Cat# HTB161
PANC1	ATCC	Cat# CRL-1469
NCI-H358	ATCC	Cat# CRL-5807
1321N1	Millipore Sigma	Cat# 86030402
A427	ATCC	Cat# HTB-53
LLC1	ATCC	Cat# CRL-1642
HCC1428	ATCC	Cat# CRL-2327
HCC1954	ATCC	Cat# CRL-2338
SiHa	ATCC	Cat# HTB-35
CaSKi	ATCC	Cat# CRL-1550
Hs695T	ATCC	Cat# HTB-137
RAW264.7	ATCC	Cat# TIB-71
HDFa	ATCC	Cat# PCS-201-012
HAEC	ATCC	Cat# PCS-100-011
A549 CAS9	Horizon	Cat#HD-CAS9-001
HCT116 CAS9	Horizon	Cat#HD-CAS9-002
MRC5	ATCC	Cat# CCL-171

**Experimental models: Organisms/strains**		

BALB/cAnNCrl	Charles Rivers Laboratories	Strain Code#028
C57BL/6NCrl	Charles Rivers Laboratories	Strain Code#027
Crl:NU*-Foxn1*^*nu*^	Charles Rivers Laboratories	Strain Code#088
NOD-*Prkdc*^*em26Cd52*^*Il2rg*^*em26Cd22*^/NjuCrl	Charles Rivers Laboratories	Strain Code#572

**Software and algorithms**		

FlowJo v.10.8.1	FlowJo	https://www.flowjo.com/
GraphPad Prism v10	GraphPad	https://www.graphpad.com/
AMI HT	Spectral Instruments Imaging	https://spectralinvivo.com/imaging-systems/
Aura 4.5.0	Spectral Instruments Imaging	https://spectralinvivo.com/software/

**Other**		

40μm Sterile Cell Strainer	Fisher Scientific	Cat#22363547
70μm Sterile Cell Strainer	Fisher Scientific	Cat#22363548
15mL conical tube	Ficher Scientfic	Cat#12565268
50mL conical tube	Fisher Scientific	Cat#12565270
gentleMACS Octo Dissociator	miltenyi biotec	Cat#130-096-427
gentleMACS C Tubes	Miltenyi biotec	Cat#1300096334
Varioskan	ThermoFisher Scientific	Cat#VLBLA0D0
Cytation 5	Aligent	Cat#CYT5MPW
LS columns	Miltenyi biotec	Cat#130-042-401
96 well flat bottom half area high binding polystyrene microplate	Corning	Cat# 3690
Nunc F96 Microwell White Polystyrene Plate	ThermoFisher Scientific	Cat#12-566-02
96 Well Assay Plate Black Plate	Corning	Cat#3603
Pierce White Opaque 96-well Plates	ThermoFisher Scientific	Cat# 15042
Falcon® 96-well Clear Round Bottom Not Treated Microplate	Falcon	Cat# 353910
Microvette 500 K3E	Starsted	Cat# 20.1341.102

### Experimental model and study participant details

#### Regulatory

Human samples were collected with Institutional Review Board approval (OP-NTD-01, OP-NTD-02). Murine studies were approved by the Institutional Animal Care and Use Committee (EB17-010-073).

#### Mice

C57BL/6 (C57BL/6N Crl), BALB/c (BALB/cAnN Crl), Nude (NU*-Foxn1*^*nu*^), and NCG (NOD-*Prkdc*^*em26Cd52*^*Il2rg*^*em26Cd22*^/Nju Crl) female mice (7–8 weeks old) were purchased from Charles River Laboratories. Male mice were excluded due to known aggression-related stress responses. Mice were housed in a specific pathogen-free (SPF) facility under controlled temperature and humidity with a 12-h light/dark cycle. Mice had *ad libitum* access to standard chow and water. Sex was determined based on supplier documentation and confirmed by visual inspection upon arrival.

#### Cell lines

Lung cancer cell lines: A427, A549, LLC1, NCI-H358, NCI-H2122, NCI-H1373, NCI-H441, and SK-LU-1; breast cancer cell lines: HCC1428, HCC1954, 4T1, E0771, and MDA-MB-231; ovarian cancer cell lines: OVCAR3; colon cancer cell lines: CT26, HT29, HCT116, and MC38; cervical cancer cell lines: SiHa and CaSki; esophageal cancer cell lines: KYSE-410; liver cancer cell lines: HepG2; head & neck cancer cells lines: FaDu and A253; pancreatic cancer cells lines: PANC1; prostate cancer cells lines: PC3; melanoma cell lines: A375, B16F10, and HS695; brain cancer cell lines: 1321N1. All cancer cells lines were purchased from ATCC except for KYSE-410 (Millipore Sigma), MC38 (Kerafast), and 1321N1 (Millipore Sigma). Macrophage cell line: RAW264.7; fibroblast cell line: human adult dermal fibroblast (HDFa); and endothelial cell line: human aortic endothelial cells (HAEC) were purchased from ATCC. Human monocytes were obtained from San Diego Blood Bank, purified, and differentiated into human monocyte-derived macrophages (HMDMs) as previously described.^48^ Briefly, monocytes were cultured in RPMI-1640 medium supplemented with 10% fetal bovine serum (FBS) and macrophage colony stimulating factor (M-CSF, 125 ng/mL, R&D Systems) for 7 days with the medium replaced every other day.

Cells were cultured in various base medias (see below), all of which were supplemented with 10% heat-inactivated FBS (Gemini Bio Products) and 1% antibiotic-antimycotic (Gibco). Base medias included: RPMI 1640 (Gibco) for NCI-H358, NCI-H2122, NCI-H1373, and NCI-H441, HCC1428, HCC1954, 4T1, OVCAR3, CT26, CaSki, KYSE-410, A549 CAS9, and HCT116 CAS9 cells; EMEM (Corning) for A427, SK-LU-1, Hs695T, HepG2, FaDu, SiHa, and MRC5 cells; DMEM (Gibco) for A549, LLC1, MDA-MB-231, E0771, PANC1, A375, B16F10, MC38, 1321N1, and RAW264.7 cells; McCoy’s 5A (Gibco) for HT29, HCT116, and A243 cells; and F-12K (Gibco) for PC3 cells.

HDFa cells were cultured in fibroblast basal medium (ATCC) supplemented with fibroblast growth kit-low serum (ATCC). Primary fibroblasts were purified from OvCa patient tumors, initially expanded in AIMV media (Gibco), and then cultured in RPMI supplemented with 10% heat-inactivated FBS (Gemini Bio Products) and 1% antibiotic-antimycotic (Gibco) for three passages prior to use in experiments. HAECs were cultured in vascular cell basal medium supplemented with endothelial cell growth kit-BBE (ATCC). Monocytes were differentiated into HMDMs by culturing in RPMI containing 10%FBS supplemented with M-CSF (125ng/mL, R&D Systems), with fresh media replacement every other day until day 7.

All cell lines were obtained directly from authenticated sources, including ATCC and other certified suppliers. Cell line identity was verified based on supplier-provided documentation, including catalog numbers, source references, and STR profiles when available. Mycoplasma contamination was routinely assessed using the MycoAlert Mycoplasma Detection Kit (IDEXX BioAnalytics), and only mycoplasma-free cultures were used. All cell lines used in this study were passaged less than 10 times before being used for experiments.

#### Primary cell isolation from OvCa patients

Primary tumor samples were obtained from female patients diagnosed with ovarian cancer (median age = 62 years; range = 34–81 years) under informed consent and approved institutional review board (IRB) protocols (OP-NTD-01, OP-NTD-02). Samples were collected at Sharp Memorial Hospital Emergency (San Diego, CA), Sharp Grossmont Hospital for Woman & Newborns (La Mesa, CA), Sharp Chula Vista Medical Center (Chula Vista, CA), Sharp Mary Birch Hospital for Woman & Newborns (San Diego, CA), and Scripps Memorial Hospital Encinitas (Encinitas, CA). Samples were collected, transported on ice, and processed (*see below*) within 24h post-collection. A total of *n* = 45 independent patient samples were analyzed. All samples were processed and analyzed under identical experimental conditions without randomization or blinding. Detailed patient clinical information, including tumor primary site, diagnostic history, disease stage and grade, and treatment history, is summarized in Table S1.

### Method details

#### Primary peripheral blood mononuclear cells (PBMCs)

Blood was collected in a CPT tube (BD Biosciences) and centrifuged at room temperature (RT) for 20min at 1600x*g*. The buffy coat (white layer) was collected, and immune cell composition was assessed by flow cytometry.

#### Primary intraperitoneal (IP) cells

IP fluid from OvCa patients was centrifuged at 400x*g* for 5min, treated with RBC lysis buffer, cells were collected, and immune cell composition was assessed by flow cytometry.

#### Primary cells from normal omental adipose tissue

Omental tissue from OvCa patients was digested with Type 1 Collagenase (1mg/mL, Worthington) in RPMI based media (ThermoFisher Scientific) at 37°C using GentleMACS Tissue Dissociator (Miltenyi Biotec) with program protocol_37C_mr_ATDK_1 to obtain stromal vascular cells (SVC). SVCs were filtered through 70μm filter (ThermoFisher Scientific), incubated in RBC lysis buffer for 5min, passed through a 40μm filter (ThermoFisher Scientific), and immune cell composition was assessed by flow cytometry.

#### Primary immune cells (IC) and cancer cells (CC) from human tumors

Tumors from OvCa patients were chopped into small pieces and digested with Type IV collagenase (Worthington) and 0.04% DNaseI (Sigma) in RPMI base media (Gibco) using GentleMACS Tissue Dissociator (Miltenyi Biotec) with program protocol_37C_h_TDK_1. Digested cells were passed through a 70μm filter (Fisher), incubated in RBC lysis buffer for 5min, and passed through a 40μm filter (ThermoFisher Scientific). CD45^+^ immune cells (IC) and CD45^−^cells were separated using CD45 microbeads (Miltenyi Biotec) following manufacture instructions. Briefly, for up to every 10 million cells, 80uL of isolation buffer was mixed with 20μL of CD45 microbeads and incubated at 4°C for 15min. Eluted cells were primarily CD45^+^ immune cells, while the flowthrough comprised mostly CD45^−^cancer cells. Cancer cells were further enriched using EPCAM microbeads if contaminated with >5% FAP+ fibroblasts. Eluted cells were primarily EPCAM+ cancer cells, while the flowthrough comprised mostly of FAP+ fibroblasts. For all samples, cell purity was assessed by flow cytometry.

#### N17350 activation

Pro-N17350 (Wuxi Biologics customized WBP7220A) is a single-point mutant of wild-type PPE (UniProt ID: P00772). The Q211F mutation was introduced by direct DNA synthesis and verified by sequencing. Active N17350 was prepared by incubating pro-N17350 (zymogen, Wuxi Biologics) with Mag-Trypsin (Takara) for 45min at 37°C in 0.1M sodium bicarbonate pH 8.0 buffer (1.25mg pro-N17350 and 2.5mL Mag-trypsin). Mag-trypsin was removed with a magnet, and the removal was validated with commercially available ELISA and enzyme activity assays. After removal, the pH was lowered to 4.0 using acetic acid, N17350 was purified and concentrated using a 3kDa centricon (Millipore Sigma), and the protein levels (A280nm) and enzymatic activity were quantified. See Figure S16 for additional details on N17350 purity and validation of its catalytic activity.

#### N17350 activity assays

Catalytic activity was measured using the fluorescence substrate AAPV-AMC (Sigma, 100μM) in a kinetic mode at 380/460nm (Varioskan LUX), and reported as initial velocity (Vo). For inactivation by PMSF, N17350 was incubated with PMSF (1mM, Sigma) for 1h at temp. Residual PMSF was eliminated with a PD-10 desalting column (GE Healthcare Life). For inactivation by plasma or human A1AT, N17350 (400nM) was incubated with human or murine plasma, or A1AT (2μM) at room temperature for 5min and the enzyme activity was measured. For inactivation by recombinant human A1AT, N17350 (5nM) was incubated with various concentrations of A1AT (0-50nM) at various AAPV-AMC substrate concentrations (0-1mM) and data were modeled according to Michaelis-Menten equations.

#### CD95 cleavage assay

Recombinant human C-terminal-CD95 (C-CD95, Wuxi Biologics) was digested with human PPE or N17350 at 1:50 enzyme:C-CD95 molar ratio for 15min at 37°C, and reactions were stopped with SDS-PAGE loading buffer. Proteins were separated on 20% SDS-PAGE gels, stained with Coomassie Blue. Gels were imaged by iBright FL1500.

#### N17350 ELISA

N17350 levels in plasma were measured using a sandwich ELISA. Polystyrene microplates were coated with anti-CELA1 antibody (LSBio, 2.5μg/mL), washed with TBST, blocked with 5% BSA/TBST for 2h, and incubated with plasma samples or recombinant N17350 (WuXi Biologics) for 2h. Wells were incubated with rabbit anti-CELA1 HRP antibody (2.5μg/mL, LSBio) for 2h, washed three times with TBST, incubated with TMB substrate (ThermoFisher Scientific) for 20min, and the signal was measured at 405nm with correction at 570nm.

#### Cell killing assays

Cancer cells or non-cancer cells were plated in complete growth media and grown to 80–90% confluence. *For N17350*, cells were washed with serum-free media (SFM), treated with various doses of N17350, and incubated at 37°C for 24h. *For chemotherapies or KRAS inhibitors*, cells were treated with various doses of drugs in complete media and incubated at 37°C for 72h. All treated cells were washed with HBSS, incubated with Calcein AM solution (C1430, Invitrogen, 4μg/mL) for 40min, washed with HBSS, and fluorescence was measured at 485/520nm (Varioskan LUX).

#### N17350 labeling with pHrodo or Alexa 680

pHrodo deep red TFP ester (ThermoFisher Scientific) or Alexa Fluor 680 NHS ester (ThermoFisher Scientific) was conjugated to N17350 or PPE following standard aminereactive ester labeling procedures. Briefly, enzyme was diluted to 1mg/mL in 0.1M sodium bicarbonate pH 8.0 and mixed with pHrodo deep red TFP ester or Alexa Fluor 680 NHS ester (dye:enzyme, 10:1 molar ratio). The reaction was incubated at room temperature for 2h with gentle shaking and unbound dye was removed using a 7 kDa Zeba desalting column (ThermoFisher Scientific) according to the manufacturer’s instructions. Dye-labeled enzymes were concentrated with an Amicon ultra centrifugal filter (Millipore sigma) and stored in PBS at −80°C until use.

#### N17350 uptake by cancer cells

CT26 cells (40,000 cells) were plated in a 96-well round-bottom plate and treated with pHrodo-labeled N17350 or PPE (200nM final) for various time points. Cells were washed twice with flow staining buffer (BD Biosciences) and cellular uptake was assessed by flow cytometry using 640nm excitation and 655 emission.

#### Mitochondrial ROS measurements

Cancer cells were treated with N17350 or ELANE (500nM, 30min), washed, labeled with the CM-H2DCFDA dye (ThermoFisher Scientific,10 mM) for 30min at 37°C, and fluorescence was quantified by flow cytometry.

#### Caspase 3/7 activity assay

Caspase-3/7 activity was measured using the Caspase-Glo 3/7 Assay Kit (Promega) per the manufacturer’s instructions. Cells were treated with N17350 or ELANE (500nM, 6h) in white 96-well plates, incubated with Caspase-Glo reagent for 30min with gentle shaking, and luminescence was measured.

#### Histone H1.0 and H1.2 knockdown

Cancer cells were transfected with Edited-R human synthetic H1.0 or H1.2 sgRNA or non-targeting control sgRNA (Dharmacon) using the CRISPR-Cas9 system according to the manufacturer’s protocol. Transfections were performed in Cas9-expressing A549 or HCT116 cells (Dharmacon) for 72 h and knockdown efficiency was assessed by immunoblotting. Cells were treated with N17350 72h post-transfection and cell viability was assessed by calcein-AM.

#### Cellular resistance and cross-resistance assays

Cancer cells were initially treated with N17350 (100nM), oxaliplatin (100μM), DOX (10μM), paclitaxel (20μM), carboplatin (100μM) and AMG-510 (1uM) and MRTX1133 (1uM) for 24-72h to produce ∼90% killing. The cells were regrown to confluence and re-treated with those cytotoxic agents for five cycles. For chemotherapy and KRAS inhibitors, higher doses were required in successive cycles to achieve 90% killing while N17350 concentrations did not require adjustment. Serially passaged non-treated cells (R0) and cells killed five successive times (R5) were treated with its corresponding cytotoxic agents to assess drug resistance. R0 and R5 cells were also treated with other cytotoxic agents in the same class to assess cross-resistance.

#### Generation of a PD-1 resistant CT26 tumor model

BALB/c female mice were inoculated with 1 × 10^6^ CT26 cells on the rear right flank and treated with anti-PD-1 (10mg/kg, i.p., days 0,3,6). Tumors were excised from mice that did not respond to anti-PD-1 therapy 10 to 14 days after the first treatment. Excised tumors were dissociated with collagenase type IV (Gibco), washed with PBS, and plated in RPMI culture media supplemented with 10% FBS (Gibco) and 1% Antibiotic-Antimycotic (Gibco). Cells were passaged at least two times, inoculated into new recipient mice, and the anti-PD-1 treatment protocol was repeated. This cycle was performed a total of six times to develop CT26 tumor-bearing mice resistant to anti-PD-1 therapy.

#### Immunogenic cell death assays

Cancer cells lines or primary cancer cells from OvCa patients were washed with SFM, treated with N17350 (500nM), oxaliplatin (100μM), or doxorubicin (10μM), and incubated at 37°C for 24h. Cell culture media were collected for ATP measurement using CellTiter Glo (G7570, Promega), while the cells were fixed in CytoFix buffer (BD Biosciences), washed, and stained with anti-CALR, anti-ANXA1, and anti-HMGB1 antibodies at RT for 15min. Cells were transferred to a 96-well U bottom plate and analyzed by flow cytometry.

#### Histone H1 translocation

Cells were plated at 40,000 cells/well in a round-bottom 96-well plate (Falcon) and treated with serum-free media (SFM) or N17350 (500nM) for 1-4h at 37°C. Cells were washed with flow staining buffer, blocked with 5% BSA, and treated with Cytofix and cytoperm buffers (to quantify cytosolic H1, BD Biosciences) or nuclear fixation and nuclear permeabilization buffers (to quantify total H1, ThermoFisher Scientific) according to manufacturer’s instructions. Fixed cells were incubated overnight at 4°C with rabbit anti-histone H1 antibody (1:250, ThermoFisher), washed, stained with PE-conjugated anti-rabbit secondary antibody (1:250, BioLegend) for 15min at RT, washed, and analyzed by flow cytometry. Histone H1 levels were quantified as mean fluorescence intensity (MFI, geometric mean) and expressed as a ratio of cytosolic/total H1.

#### Histone H1 protein levels in cells

Histone H1.0 and H1.2 levels in OvCa patient cancer cell and CD45^+^ immune cell lysates, collected in 2% SDS with protease inhibitors, were measured using a custom sandwich ELISA. Anti-H1.0 (Abcam, 2.5μg/mL) or anti-H1.2 (Proteintech, 2.5μg/mL) antibodies were coated on polystyrene microplates (Corning) or white opaque plates (ThermoFisher Scientific) respectively. After washing and blocking with 5% BSA/TBST for 2h, cell lysates or purified recombinant H1.0 or H1.2 (WuXi Biologics) were added to wells and incubated for 2h. For the H1.0 ELISA, wells were incubated with rabbit anti-H1.0 (0.1μg/mL, Sigma) for 1.5h, washed three times with TBST, incubated with HRP-conjugated anti-rabbit antibody (1μg/mL, Invitrogen) for 1h, washed with TBST, incubated with TMB substrate (ThermoFisher Scientific) for 10min, and signal was measured at 405nm with correction at 570nm. For the H1.2 ELISA, wells were incubated with biotin-conjugated anti-H1.2 antibody (0.5μg/mL, Abcam, in-house conjugated, *see below*) for 1.5h, washed with TBST, incubated with streptavidin-HRP (1:500 dilution, R&D systems) for 1h, washed with TBST, incubated with SuperSignal substrate (ThermoFisher Scientific) for 1min, and luminescence was measured. Total protein levels were quantified using a BCA assay (ThermoFisher Scientific) according to the manufacturer’s instructions. Histone H1.0 and H1.2 levels were normalized to cell lysate protein levels.

#### Biotin conjugation of antibodies

Biotin conjugation of anti-H1.2 (Abcam) was performed using biotin-NHS ester (Broadpharm) following a standard NHS conjugation protocol. Briefly, anti-H1.2 was diluted to 0.1mg/mL in sodium bicarbonate pH 8.0 and mixed with biotin-NHS ester at a 50:1 molar ratio (biotin:antibody). The reaction was incubated at room temperature for 30min with gentle mixing and quenched by adding excess glycine. Unbound biotin was removed using a 7 kDa Zeba desalting column (ThermoFisher Scientific) according to the manufacturer’s instructions. The biotin-conjugated antibody was concentrated using Amicon ultra centrifugal filter (Millipore sigma) and stored in PBS with 0.02% sodium azide at 4°C until use.

#### Tumor immunohistochemistry

Tumor microarrays (BioCoreUSA), obtained as sections with diameters of 1 mm, 1.5 mm, or 2 mm, were deparaffinized in xylene and rehydrated through graded ethanol and PBS. Antigen retrieval was performed by steaming slides in citrate buffer (DAKO target retrieval solution) for 30min, cooled to room temperature, washed with PBS, and treated with 0.3% H_2_O_2_ for 20min. Slides were then incubated with normal horse serum (Vector Laboratories) for 30min, incubated with anti-H1.0 or anti-H1.2 antibodies (Sigma, 1:100) overnight at 4°C, and signals were developed using the VECTASTAIN ABC kit (Vector Laboratories), and counterstained with hematoxylin. Images were obtained with Cytation 5 microplate reader (Agilent BioTek) at 10x magnification. For quantification, 10x scanned images were analyzed with ImageJ using color deconvolution to isolate the DAB channel, inverting the image to black-and-white, and selecting a background signal to define a threshold for identifying DAB-positive signals. A circular region of interest (ROI) corresponding to the tumor microarray core was selected, and the DAB-positive signal within this region was calculated and reported as % DAB-positive area/tumor.

#### Tumor inoculation

*For human xenograft models*, 7-8-week-old female Nude mice were inoculated with NCI-H358, NCI-H2122, NCI-H373, NCI-H441, A549, HCT116, HT29, PC3, or KYSE-410 cancer cells (5-10 × 10^6^ cells/mouse) in the right flank. *For patient-derived models*, 7-8-week-old female NCG mice were inoculated with primary cancer cells isolated from OvCa patients (5 × 10^6^ cells/mouse; right flank), or tumor tissue from colon (0.2g/tissue in 50% Matrigel; right flank) and breast cancer (0.2g/tissue in 50% Matrigel; 4^th^ mammary fat pad) patients. *For syngeneic models*, 7-8-week-old female BALB/c mice were inoculated with CT26 cells (1 × 10^6^ cell/mouse; right flank) or 4T1 cells (0.25 × 10^6^ cells/mouse; 4^th^ mammary fat pad), while 7-8-week-old C57BL6 mice were inoculated with MC38 cells (0.4 × 10^6^ cells/mouse; right flank).

#### Tumor treatments

All tumors were grown to ∼100-500mm^3^ in size prior to initiating treatments. Tumors were measured three times per week with digital calipers and tumor volume was calculated as V_T_ = (a^2^× b)/2, where a is smallest diameter and b is perpendicular diameter. Mice were euthanized using CO_2_ and underwent subsequent cervical dislocation in accordance with IACUC protocols.

##### Plasma collection

Blood (50μL, retro-orbital) was collected into EDTA-coated tubes (STARSTEDS), centrifuged at 2000*xg* for 10min, and the top plasma layer was collected.

##### Single agent studies

Tumor-bearing mice were treated with either N17350 (400μg/100mm^3^, intra-tumoral, days 0, variable), or MRTX1257 inhibitor (100mg/kg, oral, daily for 2 weeks), carboplatin (100mg/kg, i.p., days 0,7), oxaliplatin (6mg/kg, i.p., days 0,2), cyclophosphamide (100mg/kg, i.p., days 0,2), anti-CTLA-4 (5mg/kg, i.p., days 0,3,6), or anti-PD-1 (10mg/kg, i.p., days 0,3,6).

##### N17350 and CPI combination studies

Tumor-bearing mice were treated with N17350 (400μg/100mm^3^, intra-tumoral, day 0) and anti-CTLA-4 (5mg/kg, i.p., days 0,3,6), or anti-PD-1 (10mg/kg, i.p., days 0,3,6).

##### CD8^+^ T cell depletion studies

Tumor-bearing mice were treated with a high dose of anti-CD8 or IgG antibody (400μg, i.p.) before (days −3, −1) and after (day 1) N17350 treatment (400μg/100mm^3^, intra-tumoral, day 0) and a maintenance dose of anti-CD8 or IgG antibody (100μg, i.p.) once per week for uo to 3 weeks post-N17350 treatment. An identical experimental procedure was used for CD8^+^ T cell depletion in tumor rechallenge experiments (*see below*), with the exception that anti-CD8 or IgG treatments were administered pre- and post-rechallenge with CT26 cells (2 × 10^6^ cells/mouse; left flank).

##### Tumor rechallenge studies

For rechallenge studies, CT26 tumor-bearing mice that became tumor-free following N17350 treatment (400μg/100mm^3^, intra-tumoral, day 0) were re-inoculated with CT26 cells 90 days (0.25 × 10^6^ cells), 150 days (2 × 10^6^ cells), and 400 days (2 × 10^6^ cells) after treatment with N17350. The final rechallenge was performed in mice treated with anti-CD8 or IgG antibodies as described above.

For dual flank studies, ‘CT26-cured’ and ‘MC38-cured’ mice – those made tumor free with a single intra-tumoral treatment with N17350 (400μg/100mm^3^, day 0) – were re-inoculated with CT26 or MC38 cells respectively in the left flank and challenged with 4T1 or B16F10 cells respectively in the right flank (0.25 × 10^6^ cells) 90 days after treatment with N17350.

#### Flow cytometry studies

All flow cytometry data were acquired on a BD FACSymphony Flow Cytometer (BD Biosciences) and data were analyzed with FlowJo software (v.10.8.1).

##### Murine studies

Tumors were excised, weighed, and digested for 60min at 37°C with digestion buffer: DNaseI (40 U/mL; Sigma-Aldrich), Hyaluronidase (60 U/mL; Sigma-Aldrich), Collagenase type IV (4U/mL; Gibco) in RPMI media. Cells were filtered through a 70μm cell strainer (BD Falcon), washed, resuspended in RPMI media, and stained with monoclonal antibodies at 4°C for 30min.

##### CD8^+^ T cell AH-1 dextramer quantification

Blood, collected from CT26 or 4T1 tumor-bearing mice treated with N17350 (400μg, intra-tumoral, day 0) or PBS was diluted with PBS (1:1 ratio), treated with red cell lysis buffer for 5min at room temperature, and cells were collected by centrifugation at 300*xg* for 5min. Cell pellets were resuspended in AH-1 specific PE-labeled dextramer (H-2L^d^ SPSYVYHQF) for 30min at 4°C, and stained with a T cell specific antibody cocktail according to the manufacturer’s instructions.

##### Human studies

Purified human populations were stained with monoclonal antibodies and cell viability stain L/D aqua at RT for 15min, washed, resuspended in FACS buffer, and subject for flow cytometry analysis.

#### TCGA analysis

Analysis of histone H1 levels in tumor and adjacent normal tissues was conducted using the TIMER 2.0 database (http://timer.cistrome.org/), which provides RNA-seq expression data from The Cancer Genome Atlas (TCGA). The “Gene Expression” module was used, and *HIST1H1C* or *H1F0* was input to assess expression. Boxplots displayed the distribution of gene expression levels, and differential expression between tumor and normal tissues were evaluated using the Wilcoxon test. Figure 7A summarizes the up- or down-regulation of histone H1 in tumors compared to normal tissues across various cancer types.

#### N17350 tumor pK quantification

CT26 tumor-bearing mice (∼100mm^3^) were anesthetized and injected with Alexa 680-labelled N17350. Tumors were imaged in live mice with a spectral Ami HT live animal imager (Spectral instrument imaging, Tuscon, AZ, USA). Luminescence intensity was quantified as photon counts per second using the Aura4.5.0 Image software (Spectral).

### Quantification and statistical analysis

Statistical analyses were performed using Prism 10.1.2 GraphPad (GraphPad software) represented by NS no significant difference; ∗*p* < 0.05, considered statistically significant.

Statistical details for all experiments, including the statistical tests used, exact values of n, definitions of n, measures of central tendency, and measures of variability are provided in the figure legends.

All data represent independent biological replicates and are shown as mean ± SEM. EC_50_ values were determined by nonlinear regression using a four-parameter variable slope model (GraphPad Prism v10). For immune or non-cancer cells lacking a response plateau, EC_50_ values were assigned as >10,000 nM.

For tumor growth data analysis, mixed effects two-way ANOVA was used to compare the average tumor growth rates among groups by assessing the significance of the treatment by time interaction. Survival analyses were performed using the Mantel–Cox (log rank) test to evaluate differences between groups.

Flow cytometry data were acquired using a BD Symphony flow cytometer (BD Biosciences) and analyzed with FlowJo software v10.9 (BD Biosciences), gating was established with FMO controls and compensation was performed using single-stained controls, and all samples were processed under consistent instrument settings.

## Supplemental information

Document S1. Figures S1–S16

Document S2. Article plus supplemental information
