# Supplementary material for: Developing a therapeutic elastase that stimulates anti-tumor immunity by selectively killing cancer cells
Source: Cell Rep Med. 2025 Nov 7;6(11):102446. doi: 10.1016/j.xcrm.2025.102446 (PMC12711694; doi:10.1016/j.xcrm.2025.102446)
Supplement: Document S1. Figures S1–S16 [file mmc1.pdf]

Cell Reports Medicine, Volume *6*

Supplemental information

Developing a therapeutic elastase that stimulates anti-tumor immunity by selectively killing cancer cells

Ravindra Gujar, Chang Cui, Maria Fumagalli, Nicole Martinez, Afshin Bahador, Alain Algazi, Kevin Harrington, Court Turner, and Lev Becker

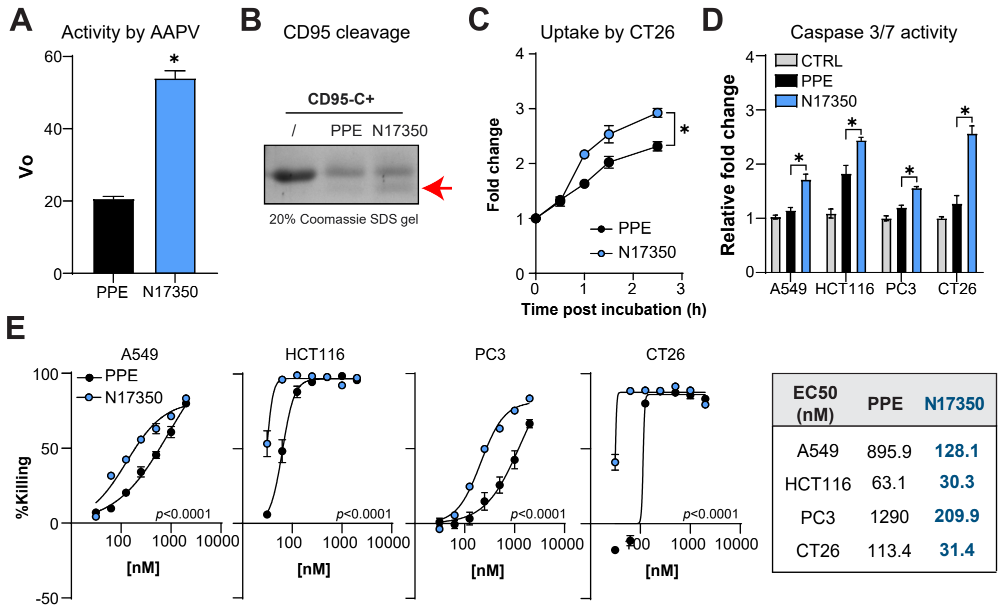

Figure S1. Comparing the enzymatic and cancer killing properties of N71350 to PPE (Related to all figures). _Panel A_: Enzymatic activity (initial rate, Vo) based on cleavage of an AAPV fluorogenic substrate. _Panel B_: CD95 cleavage assessed by SDS-PAGE and Coomassie Blue staining (1:50 enzyme:substrate molar ratio, 15min). Red arrow denotes the C-terminal death-domain (DD) of CD95. _Panel C_: Uptake of pHrodo-labeled N17350 and PPE (200nM) by CT26 cells (n=3/group) was assessed by flow cytometry. _Panel D_: Caspase-3/7 activity post treatment with N17350 or PPE (500nM, 6h). Data were normalized to serum-free media (SFM) control (n=3/group). _Panel E_: Cancer cell killing curves (*left*) and EC50 values (*right*) for N17350 and PPE (6h). *, $p<0.05$, Student's t-test (*A*), two-way ANOVA (*C,D*), extra sum-of-squares F test (*E*). Results are mean ± SEM. All replicates are independent biological replicates.

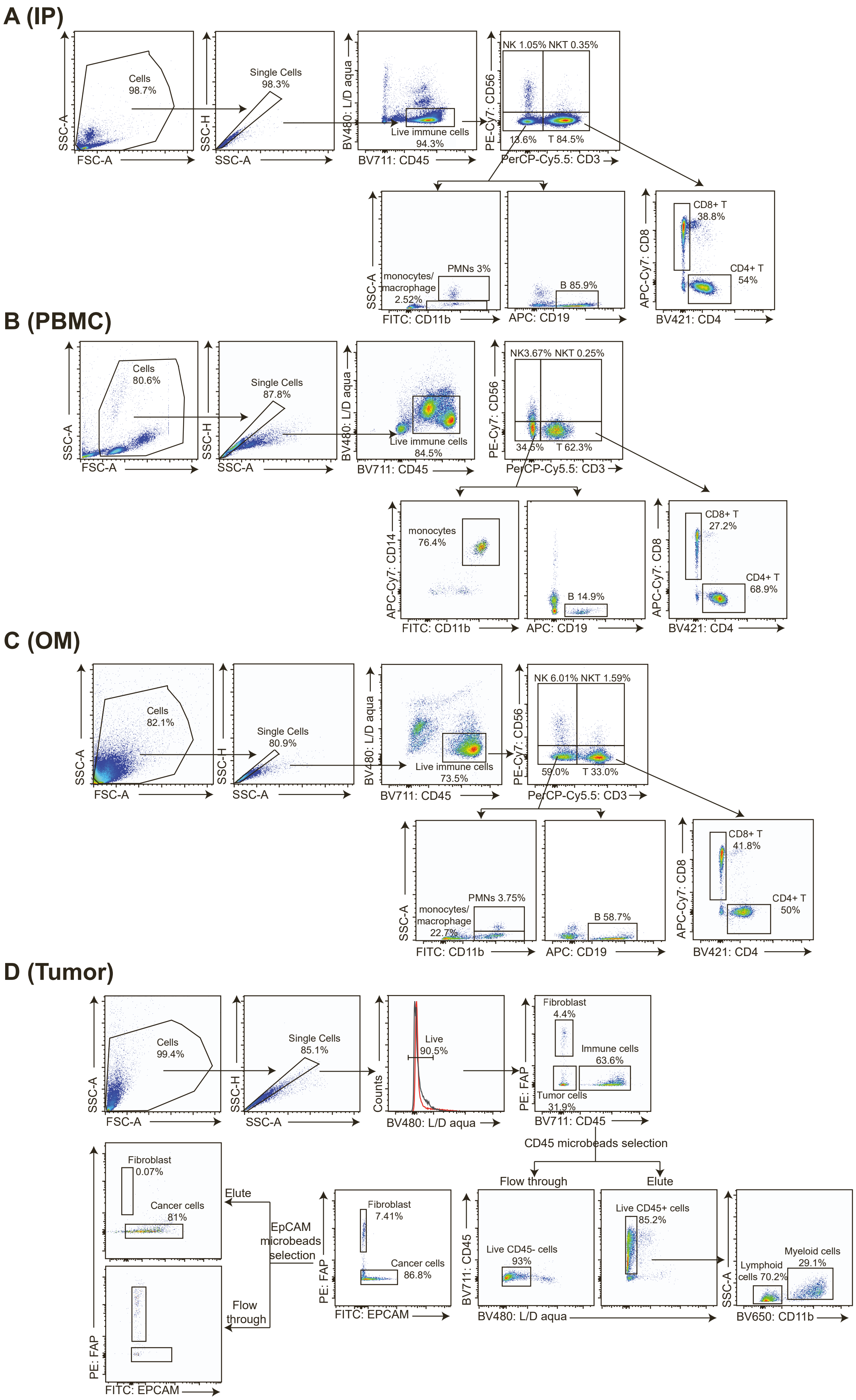

Figure S2. Cellular compositions in samples collected from OvCa patients (Related to figures 1,2,6,7). *Panels A-C*: Intraperitoneal (IP) fluid, normal omental adipose tissue (OM), and PBMCs were isolated from OvCa patients and cellular composition was determined by flow cytometry. *Panel D*: Purification of cancer cells, CD45+ immune cells, and fibroblasts from tumors of OvCa patients was assessed by flow cytometry.

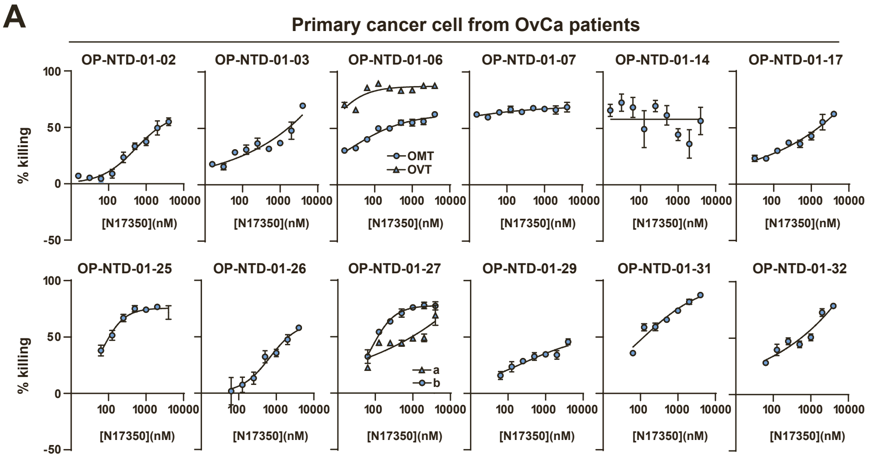

C

Patient ID	Tumor Location	Cancer Cells EC50 (nM)	Immune Cells EC50 (nM)
OP-NTD-01-02	Ovary	536.3	>10000
OP-NTD-01-03	Omentum	550.7	
OP-NTD-01-04	Omentum	481.1	>10000
OP-NTD-01-06a	Ovary	7.8	
OP-NTD-01-06b	Omentum	20.61	
OP-NTD-01-07	Fallopian tube	7.8	
OP-NTD-01-14	Ovary	7.8	
OP-NTD-01-17	Gastric	730.4	
OP-NTD-01-20	Peritoneal	59.73	
OP-NTD-01-22	Omentum	121.1	>10000
OP-NTD-01-24	Pelvic/Ovary	540.6	>10000
OP-NTD-01-25	Ovary	65.43	>10000
OP-NTD-01-26	Omentum	675.3	
OP-NTD-01-27a	Ovary	115.9	>10000
OP-NTD-01-27b	Omentum	76.01	>10000
OP-NTD-01-29	Omentum	235.6	
OP-NTD-01-31	Ovary	114	
OP-NTD-01-32	Omentum	684.4	

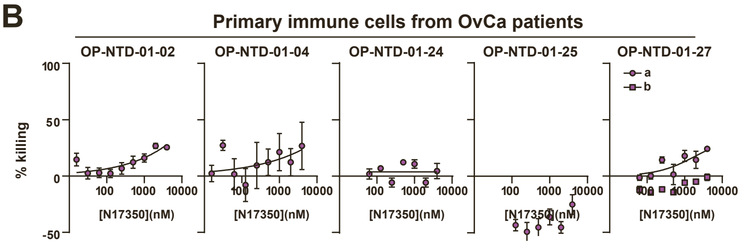

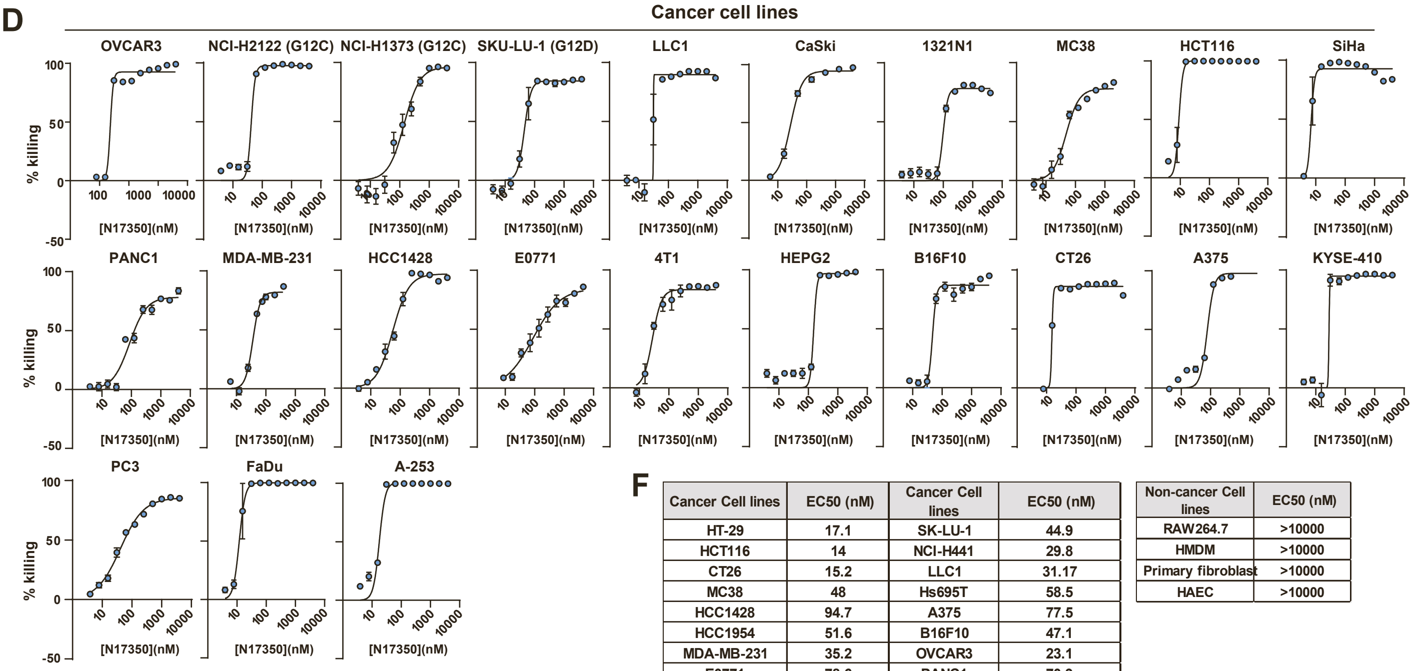

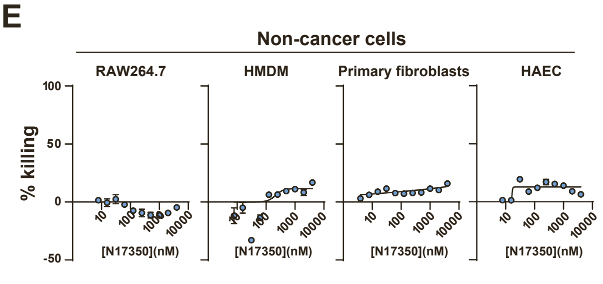

F

Cancer Cell lines	EC50 (nM)	Cancer Cell lines	EC50 (nM)
HT-29	17.1	SK-LU-1	44.9
HCT116	14	NCI-H441	29.8
CT26	15.2	LLC1	31.17
MC38	48	Hs695T	58.5
HCC1428	94.7	A375	77.5
HCC1954	51.6	B16F10	47.1
MDA-MB-231	35.2	OVCAR3	23.1
E0771	78.6	PANC1	70.9
4T1	27.7	PC3	36
HepG2	140.9	FadDu	12
NCI-H358	29.2	A253	17.8
A549	20.9	SiHa	10
NCI-H2122	42.3	CaSKi	27.4
NCI-H1373	142.5	1321N1	98.2
A-427	54.8	KYSE-410	28.2

Non-cancer Cell lines	EC50 (nM)
RAW264.7	>10000
HMDM	>10000
Primary fibroblast	>10000
HAEC	>10000

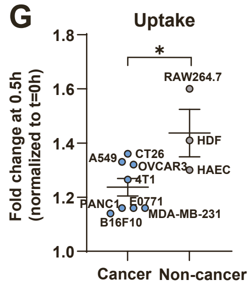

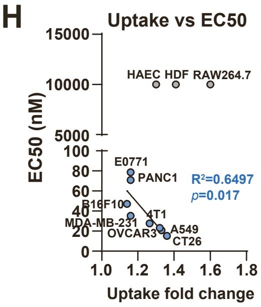

Figure S3. N 17350 killing curves for cancer cells (Related to figures 1,6). Cancer cells were treated with N17350 for 24 hours, and cell viability was measured using calcein AM. EC50 values were determined via non-linear regression (n=3-6/dose). _Panel A_: Primary cancer cells from OvCa patients. _Panel B_: Primary immune cells from OvCa patients. Panel C: EC50 summary table for primary cells. _Panel D_: Cancer cell lines. _Panel E_: Non-cancer cells. _Panel F_: EC50 summary table for cancer and non-cancer cells. _Panel G_: Uptake of pHrodo-labeled N17350 (200nM) by cancer and non-cancer cells was assessed by flow cytometry. Data was shown as 30 min signal normalized to t=0 (fold change). *, $p<0.05$, Student's t-test. _Panel H_: Correlation between pHrodo uptake and N17350 EC50 across cell types. Linear fit was applied to cancer cell data. Results are presented as mean ± SEM. _Note_: Immune cell EC50 values could not be reliably estimated from plateau-based models and were conservatively set to >10,000 nM. All replicates are independent biological replicates.

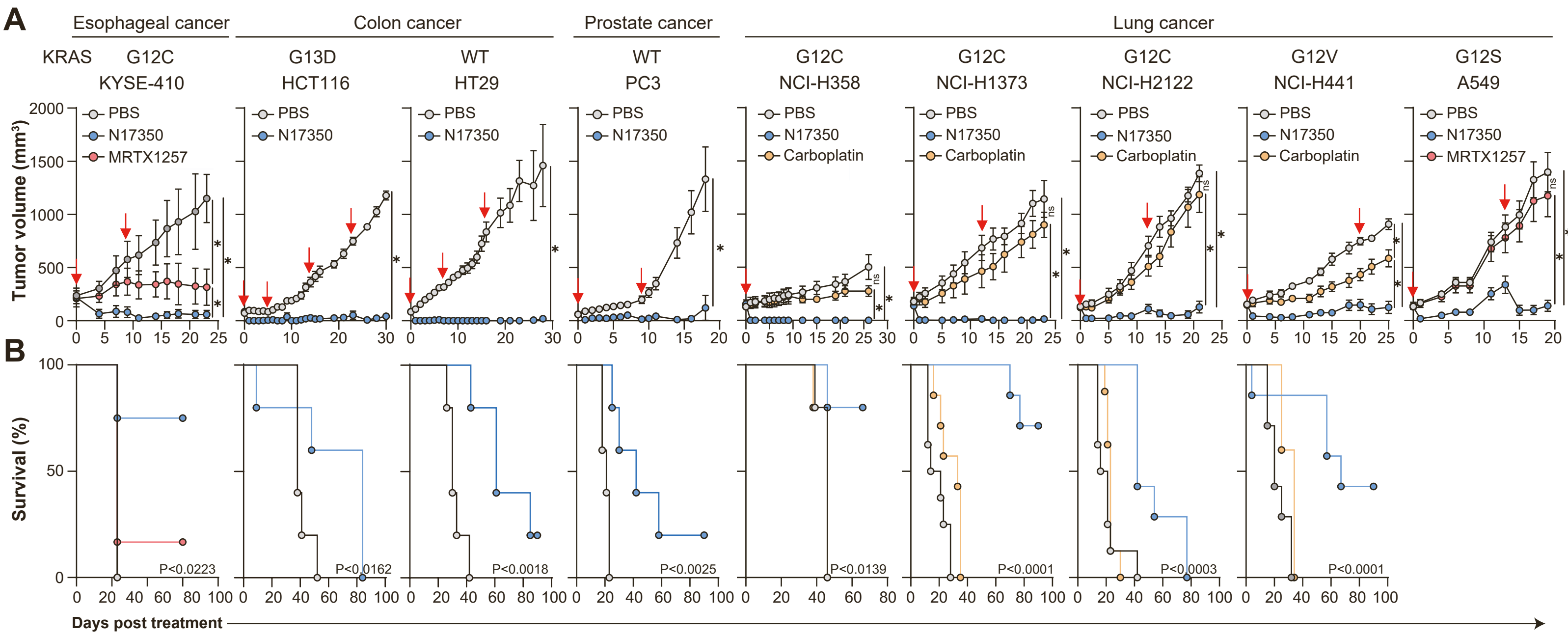

Figure S4. N17350 exhibits pan-cancer efficacy and outperforms standard-of-care therapies (Related to figure 1). Tumor-bearing mice were treated with N17350 (400 µg/100mm³, intra-tumoral;), MRTX1257 (100mg/kg, oral, daily), or carboplatin (100mg/kg, intraperitoneal, days 0,7); n=5 mice/group. _Panel A_: Tumor growth curves. _Panel B_: Overall survival plots. * $p<0.05$, two-way ANOVA; Mantel-Cox test (survival). Results are mean ± SEM. Arrows indicate N17350 treatment. All replicates are independent biological replicates.

A

Murine Cancer Cells

Marker	N17350	OXP	DOX
ANXA1	**4/4**	1/4	1/4
HMGB1	**4/4**	1/4	4/4
CALR	**4/4**	3/4	0/4
ATP	**4/4**	2/4	1/4

C

Human Cancer Cells

Marker	N17350	OXP	DOX
ANXA1	**2/2**	1/2	1/2
HMGB1	**2/2**	0/2	2/2
CALR	**0/2**	2/2	0/2
ATP	**2/2**	1/2	2/2

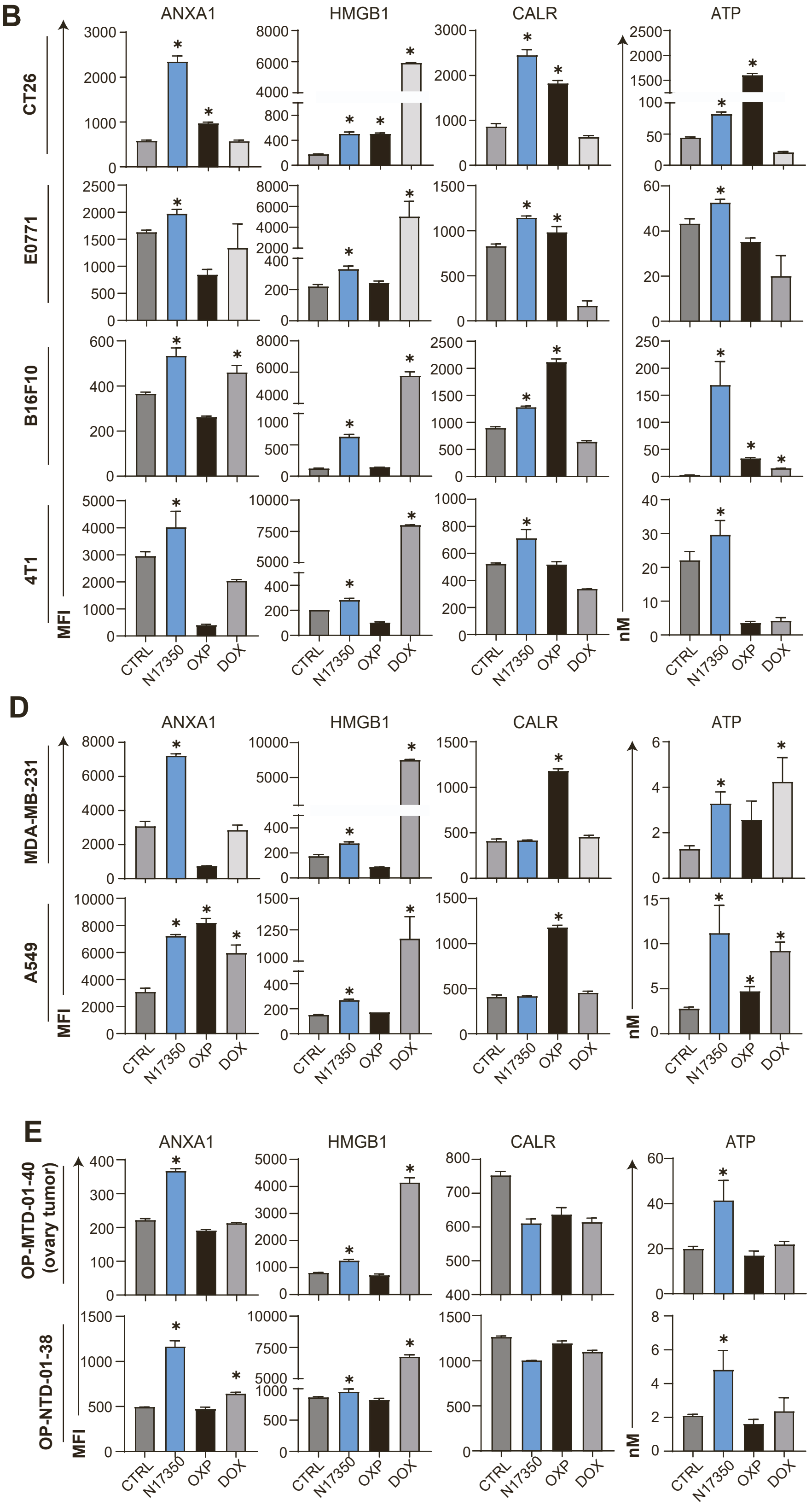

Figure S5. N17350 induces ICD in human and murine cancer cells (Related to figure 2). Murine (B16F10, E0771, 4T1, CT26) or human (MDA-MB-231, A549) cancer cells or primary cancer cells isolated from OvCa patients (OP-NTD-01-40 (ovary tumor) and OP-NTD-01-38) were treated with N17350 (500nM), oxaliplatin (OXP; 100µM), or doxorubicin (DOX; 10µM) for 24h and ICD markers were measured; n=3/group. _Panels A,C_: Summary of ICD markers across all murine cancer cell lines (*Panel A*) and human cancer cell lines (*Panel C*) tested (number of cell types elevated/tested). _Panels B,D,E_: ICD marker quantification in murine cancer cell lines (*Panel B*), human cancer cell lines (*Panel D*), and primary cancer cells isolated from OvCa patients (*Panel E*). * $p<0.05$, Student's t-test. Results are mean ± SEM. All replicates are independent biological replicates.

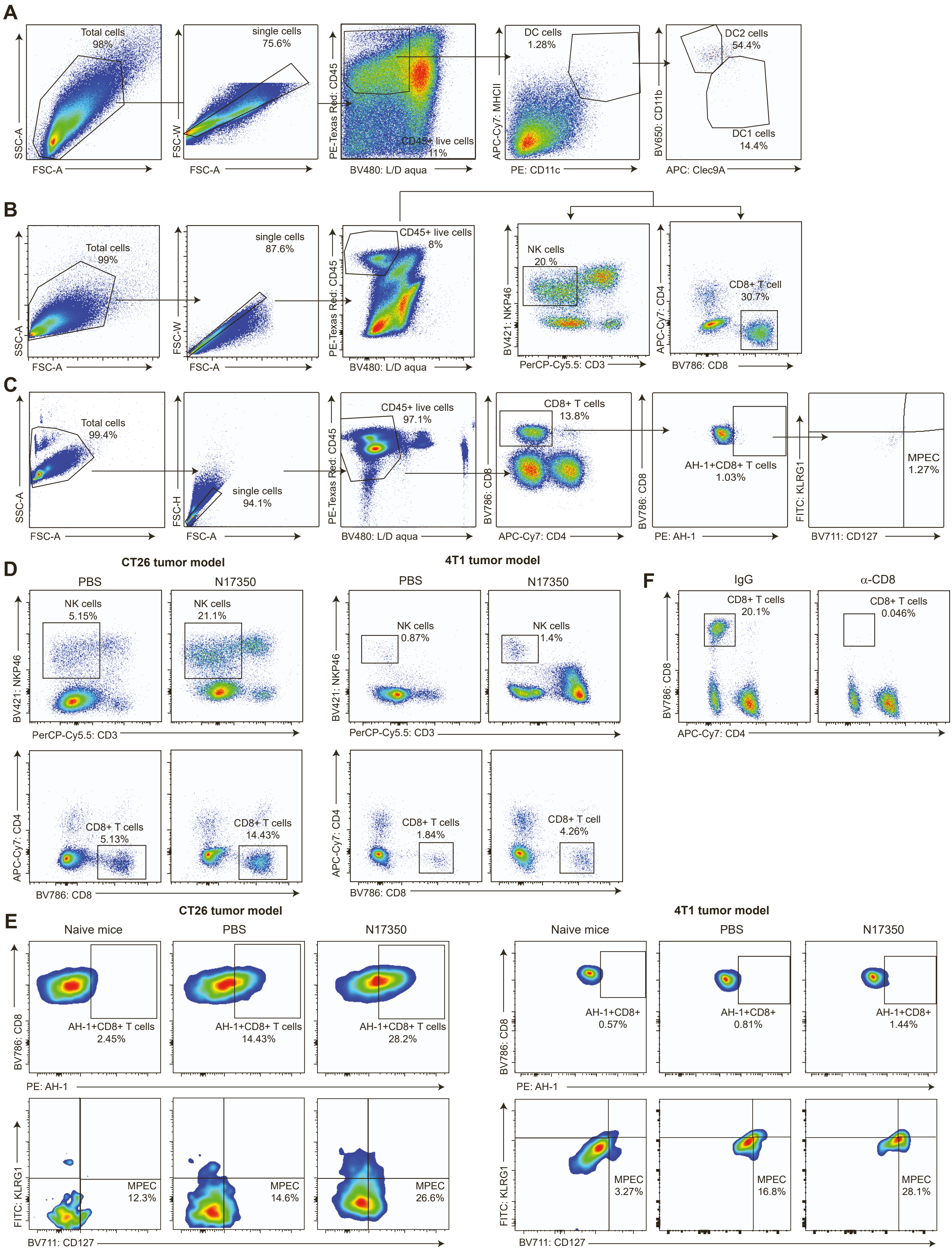

Figure S6. Representative flow cytometry analyses in the CT26 and 4T1 models (Related to figures 2,3). Panels A-C: Schematic representation of gating strategies for myeloid (*Panel A*) and lymphoid (*Panel B*) cells in the tumor, and AH-1 dextramers in blood (*Panel C*). Panels D-E: CT26 (100µg, intra-tumoral) or 4T1 (400µg, intra-tumoral) tumor-bearing mice were treated with a single dose of N17350 and immune profiles were assessed in the tumor (*Panel D*) and blood (*Panel E*) 12 days after treatment. *Panel F*: Representative data to validate $CD8^{+}$ T cell depletion in mice. Blood $CD8^{+}$ T cell depletion in blood was validated prior to treatment with N17350 or rechallenge of N17350 cured mice.

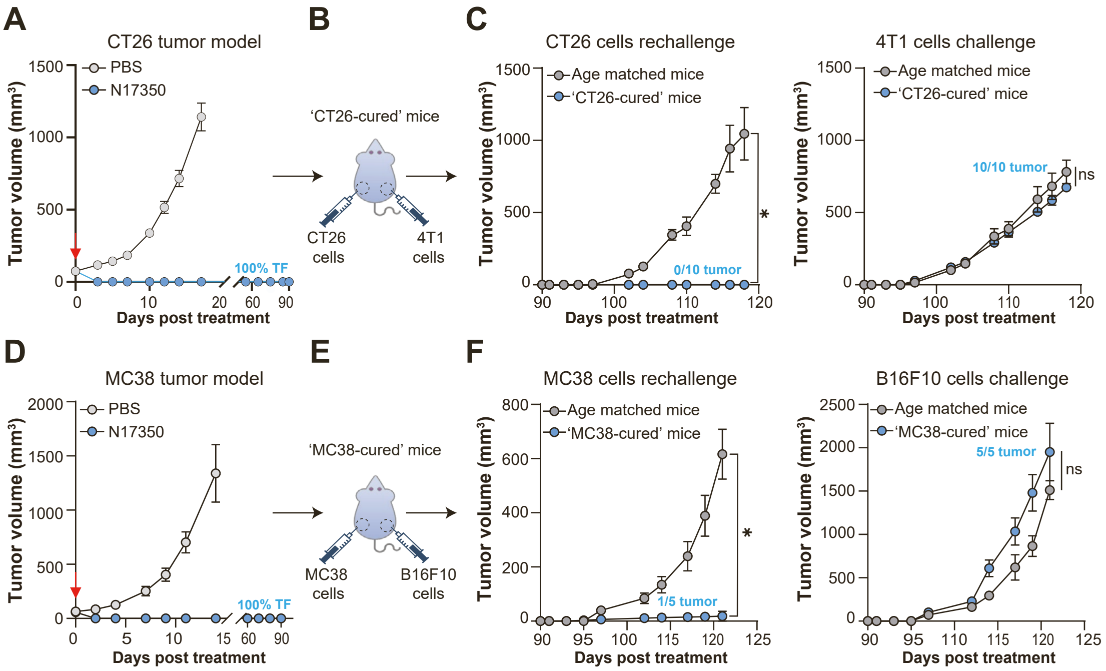

Figure S7. N17350 induces tumor-specific immune memory (Related to figure 3). Panel A,D: Tumor-bearing BALB/C mice (CT26 model) or C57BL/6 mice (MC38 model) were treated with N17350 (400 µg/100mm3, intra-tumoral, day 0); n=5-10 mice/group. Generation of 'CT26-cured' mice (*Panel A*) and 'MC38-cured' mice (*Panel D*) with a single injection of N17350. Panels B,E: Schematic of dual flank tumor challenge of 'CT26-cured' mice (*Panel B*) and 'MC38-cured' mice (*Panel E*). Panels C,F: 'CT26-cured' mice (*Panel C*) and 'MC38-cured' mice (*Panel F*) were challenged 90 days post N17350 treatment with CT26 or MC38 ($0.25x10^6$ cells, *left flank*) and syngeneic but immunologically distinct 4T1 or B16F10 cell respectively ($0.25x10^6$ cells, *right flank*) on the opposing flank. *, $p<0.05$, two-way ANOVA. Results are mean ± SEM. Arrows indicate N17350 treatment. All replicates are independent biological replicates.

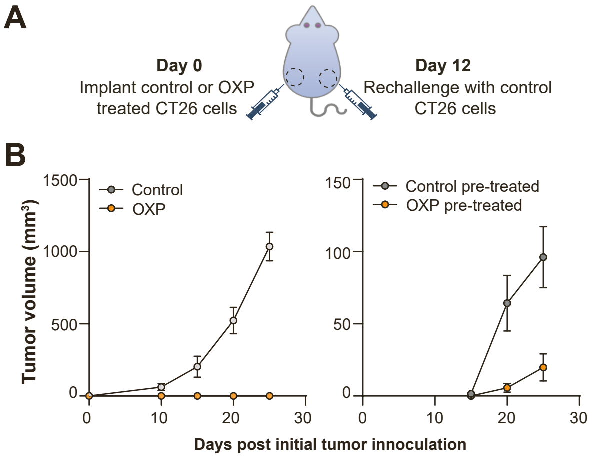

Figure S8. Oxaliplatin induces anti-tumor immunity in a standard immunogenic cell death assay (Related to figure 2). _Panel A_: Control (serum-free media, 4h) and oxaliplatin (OXP)-treated (100μM, 4h) CT26 cells were implanted into the right flank (1×10^6 cells) of BALB/c mice. Untreated CT26 cells (1×10^6) were implanted in the left flank 12 days later. _Panel B_: Tumor growth was monitored at the primary (*left*) and secondary sites (*right*); n=5/group. All replicates are independent biological replicates.

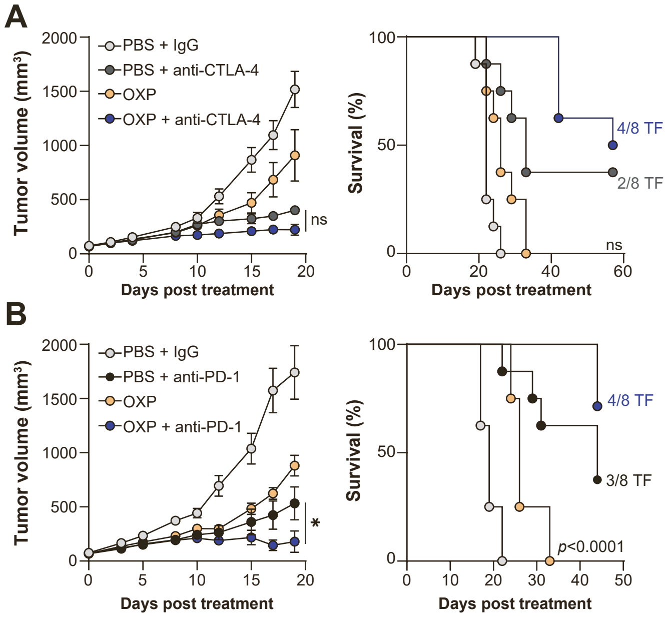

Figure S9. Oxaliplatin and CPI combination therapy in the CT26 model (Related to figure 4). Panel A: Effects of oxaliplatin (OXP; 6mg/kg, i.p., days 0, 2) and anti-CTLA-4 (5mg/kg, i.p., days 0, 3, 6), alone or in combination, in the CT26 model. Primary tumor growth (*left*), and overall survival (*right*); n=8 mice/group. Panel B: Effects of oxaliplatin (6mg/kg, i.p., days 0, 2) and anti-PD-1 (10mg/kg, i.p., days 0, 3, 6), alone or in combination, in the CT26 model. Primary tumor growth (*left*), and overall survival (*right*); n=8 mice/group. * $p<0.05$, two-way ANOVA; Mantel-Cox test (survival). Results are presented as mean ± SEM. All replicates are independent biological replicates.

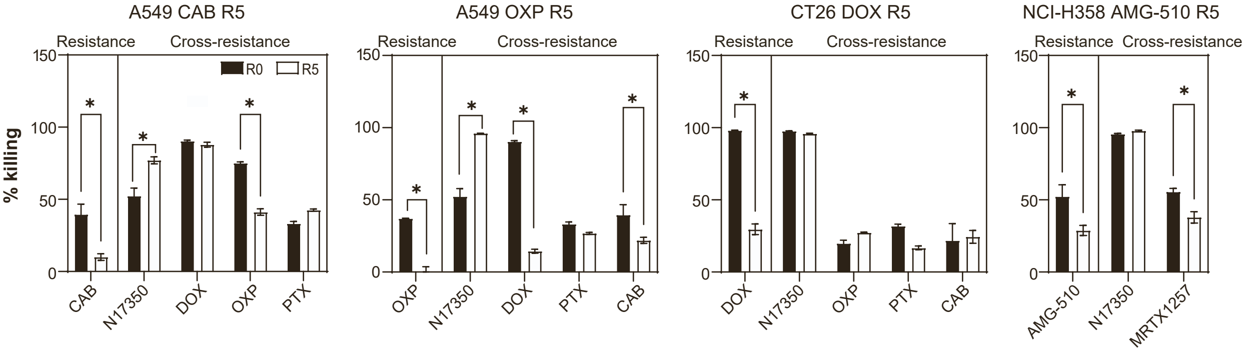

Figure S10. N17350 avoids cross-resistance to chemotherapies and KRAS inhibitors (Related to figure 6). Previously established control (R0) and chemotherapy- or KRAS inhibitor-resistant (R5) cells (*see Fig. 5*) were treated with their corresponding drug to confirm resistance. To evaluate cross-resistance, R0 and R5 cells were treated with N17350 or other drugs from the same class. Cells were treated with N17350 (500nM, 24h), paclitaxel (PTX, 0.6µM, 72h), doxorubicin (DOX, 1.9µM, 72h), oxaliplatin (OXP, 33.3µM, 72h), carboplatin (CAB, 66.7µM, 72h), AMG-510 (82µM, 72h), MRTX1257 (206µM, 72h) and cell viability was assessed; n=3/group. * p<0.05, two-way ANOVA. Results are presented as mean ± SEM. All replicates are independent biological replicates.

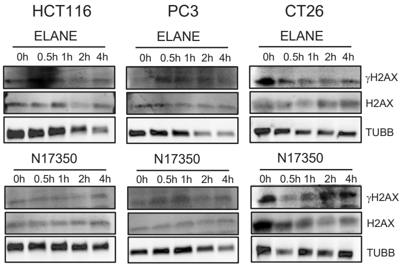

Figure S11. N17350 targets the ELANE pathway (Related to figure 7). Immunoblots of γH2AX/H2AX ratio (DNA damage marker) post treatment with N17350 or ELANE (200nM, 0-4h). For quantification, *see Fig 7B*.

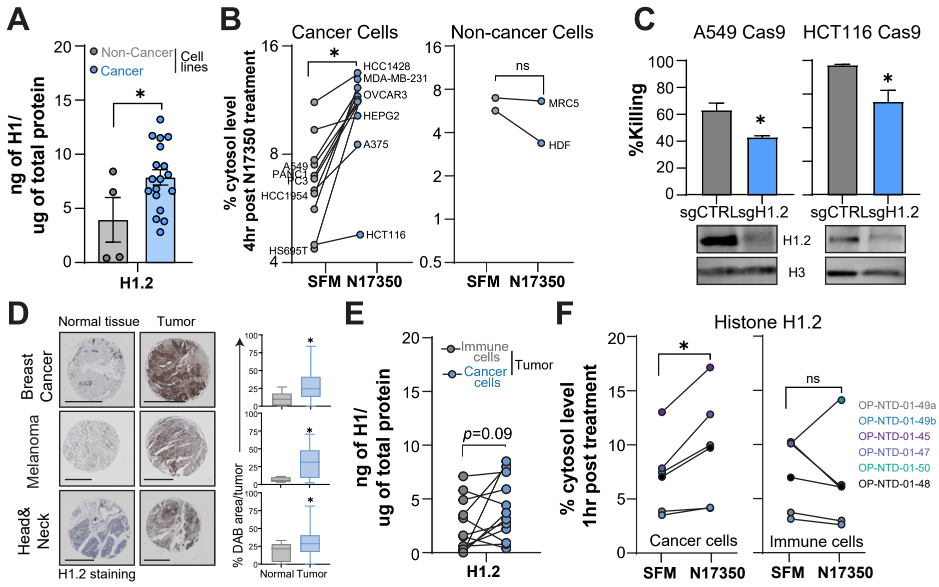

Figure S12. Involvement of histone H1.2 in N17350-mediated selective cancer cell killing (Related to figure 7). _Panel A_: Histone H1.2 levels in cancer and non-cancer cell lines were measured by ELISA and normalized to total cell protein. _Panel B_: Cytosolic translocation of histone H1.2 post N17350 (200nM, 4 h) or serum-free media (SFM) treatment was quantified by flow cytometry and normalized to total H1.2. _Panel C_: Effect of H1.2 knockdown on N17350 (31.3nM, 6h) killing of A549 Cas9 and HCT116 Cas9 cells (*left*) and validation of knockdown efficiency at 72h (*right*); n=3/group. _Panel D_: Tumor microarrays from melanoma, breast, and head and neck cancer patients were stained with anti-H1.2 antibodies, using normal tissue as a control. Representative images (*left*) and quantification (*right*). Scale bar = 1mm. _Panel E_: H1.2 levels in primary cancer cells and CD45+ immune cells isolated from the same tumor of OvCa patients. _Panel F_: Effect of N17350 (500nM, 1h) on histone H1.2 cytosolic translocation in primary cancer cells or CD45+ immune cells from tumors or intraperitoneal fluid of the same patient. *, $p<0.05$, Student's t-test: unpaired (*A,C,D*) and paired (*B,E,F*). Results are mean ± SEM. All replicates are independent biological replicates.

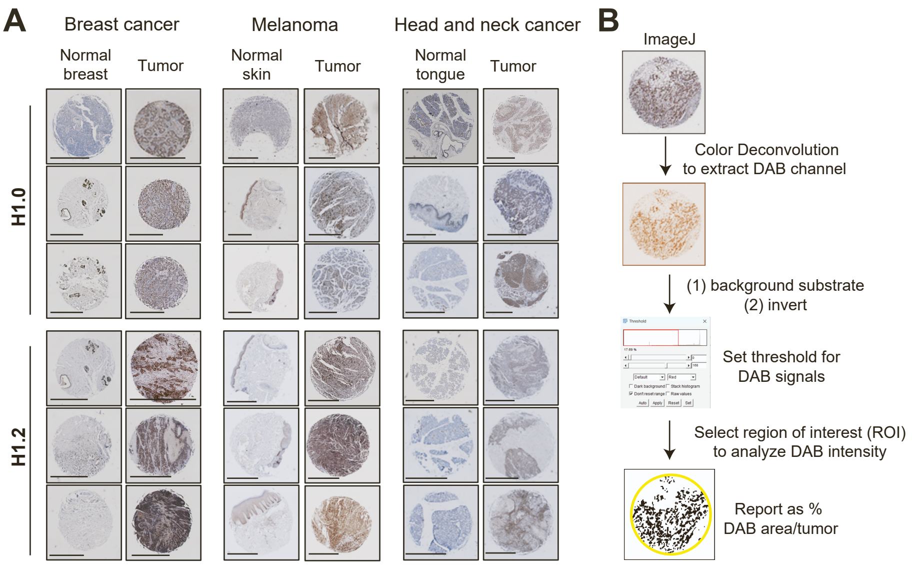

Figure S13. Representative IHC images of histone H1.0 and H1.2 across patient tumor types (Related to figure 7). *Panel A*: Tumor microarrays from melanoma, head and neck, and breast cancer patients were stained with antibodies against human histone H1.0 or H1.2. Each panel represents one patient sample. Scale bar = 1mm. *Panel B*: Method for quantifying histone H1.0 and H1.2 levels in tissues based on IHC staining (*see Figs. 7I, S12D*).

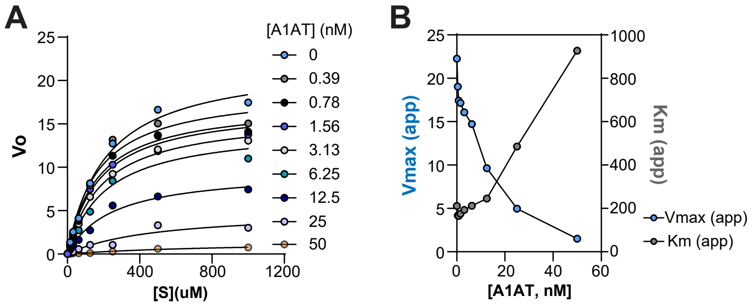

Figure S14. A1AT inhibits N17350 through a mixed-mode mechanism (Related to figure 3). *Panel A*: N17350 (5nM) was incubated with various concentrations of A1AT at various substrate concentrations and catalytic activity was measured. Km(app) and Vmax(app) values (symbols) were obtained by fitting curves to Michaelis-Menten equations (lines). *Panel B*: Km(app) and Vmax(app) versus A1AT plots are consistent with a mixed-mode inhibition mechanism. Global modeling of data in panel A with a mixed-mode inhibition mechanism estimated an inhibition constant (Ki) of 6.4nM. All replicates are independent biological replicates.

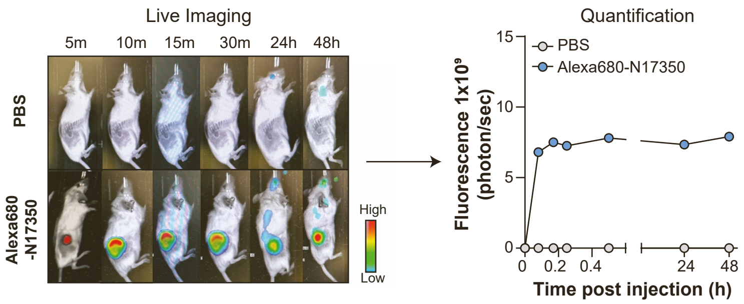

Figure S15. N17350 tumor pharmacokinetics (Related to all figures). CT26 tumor-bearing mice were injected intra-tumorally with Alexa680-labeled N17350 (5µg) and tumor pK was assessed over a 48h period by bioluminescence imaging. Mouse tumor imaging (*left*) and quantification (*right*). Note: A sub-therapeutic dose of N17350 was used because therapeutic doses produce rapid tumor regression complicating an assessment of tumor pK.

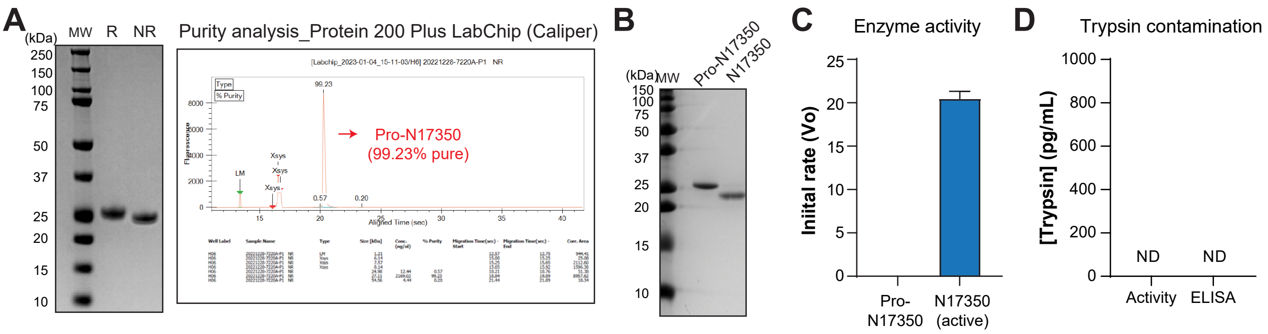

Figure S16. N17350 activation and purification (Related to all figures). _Panel A_: Pro-N17350 purity assessed by SDS-PAGE and Coomassie Blue staining under reducing (R) and non-reducing (NR) conditions (*left*) and by Protein 200 Plus LapChip analysis (*right*). _Panels B-D_: Pro-N17350 was converted to N17350 by incubation with trypsin. Proteolytic cleavage was confirmed by SDS-PAGE and Coomassie Blue staining (*Panel B*), enzyme activity was confirmed with a fluorescent peptide substrate activity assay (*Panel C*), and trypsin removal was confirmed by a trypsin catalytic activity assay and by ELISA (*Panel D*). ND = not detected. Results are mean ± SEM. All replicates are independent biological replicates.
